# Genetic Evidence in Favor of a Polyketide Origin of Acremeremophilanes, the Fungal “Sesquiterpene” Metabolites

**DOI:** 10.1128/spectrum.01793-22

**Published:** 2022-08-08

**Authors:** Ravindra Bansal, Sunil Kumar Sethy, Zareen Khan, Nasiruddin Shaikh, Kaushik Banerjee, Prasun K. Mukherjee

**Affiliations:** a Nuclear Agriculture and Biotechnology Division, Bhabha Atomic Research Centre, Mumbai, Maharashtra, India; b National Referral Laboratory, ICAR–National Research Centre for Grapes, Pune, Maharashtra, India; University of Michigan

**Keywords:** *Trichoderma*, secondary metabolism, acremeremophilane, gene cluster, biosynthesis, *Stachybotrys*

## Abstract

Eremophilanes are a large group of “sesquiterpenes” produced by plants and fungi, with more than 180 compounds being known in fungi alone. Many of these compounds are phytotoxic, antimicrobial, anticancer and immunomodulators, and hence are of great economic values. Acremeremophilanes A to O have earlier been reported in a marine isolate of *Acremonium* sp. We report here the presence of Acremeremophilane I, G, K, N, and O, in a plant beneficial fungus *Trichoderma virens*, in a strain-specific manner. We also describe a novel, P strain-specific polyketide synthase (PKS) gene cluster in *T. virens*. This gene cluster, designated *amm* cluster, is absent in the genome of a Q strain of *T. virens*, and in other *Trichoderma* spp.; instead, a near identical cluster is present in the genome of the toxic mold Stachybotrys chartarum. Using gene knockout, we provide evidence that acremeremophilanes are biosynthesized via a polyketide route, and not via the mevalonate/terpene synthesis route as believed. We propose here that the 10-carbon skeleton is a product of polyketide synthase, to which a five-carbon isoprene unit is added by a prenyl transferase (PT), a gene for which is present next to the PKS gene in the genome. Based on this evidence, we propose that at least some of the eremophilanes classified in literature as sesquiterpenes (catalyzed by terpene cyclase) are actually meroterpenes (catalyzed by PKSs and PTs), and that the core moiety is not a sesquiterpene, but a hybrid polyketide/isoprene unit.

**IMPORTANCE** The article contradicts the established fact that acremeremophilane metabolites produced by fungi are sesquiterpenes; instead, our findings suggest that at least some of these well-studied metabolites are of polyketide origin. Acremeremophilane metabolites are of medicinal significance, and the present findings have implications for the metabolic engineering of these metabolites and also their overproduction in microbial cell factories.

## INTRODUCTION

The filamentous fungi *Trichoderma* spp. are widely used in agriculture as plant growth promoters and biofungicides (1–4). Plant beneficial effects of these microbes have been attributed to their ability to kill/inhibit other fungi, to boost plant immunity, and to the production of a large array of secondary (specialized) metabolites (5–10). About 400 small molecule secondary metabolites (mainly nonribosomal peptides, polyketides, and terpenes) have been reported from *Trichoderma* spp., in addition to more than 1000 peptaibols (5, 7). *Trichoderma virens* is one of the most researched species for genetics, genomics, and development of commercial formulations (11–13). Interestingly, *T. virens* population exist as two strains, classified based on their ability to produce secondary metabolites (14). The P strains produce gliovirin, while the Q strains produce gliotoxin (both nonribosomal peptides). Both P and Q strains produce viridin, viridiol (steroids), heptelidic acid (sesquiterpene), and several volatile sesquiterpene metabolites (14–17). The biosynthesis gene clusters for all these metabolites have been revealed by genome analysis and gene deletion experiments (18–21). As with other fungi, the *Trichoderma* genome analysis revealed that the biosynthesis potential far exceeds that of the metabolites reported, as most gene clusters are silent under standard laboratory cultivation conditions (5, 8). The genome of *T. virens* Q strain (Gv29-8) was already available in the database (22). We earlier sequenced the genome of a P strain, i.e., IMI 304061 (19). Comparing these two genome sequences, we found a PKS cluster that is unique to P strain, and has similarity with a gene cluster from Stachybotrys chartarum, rather than with any other *Trichoderma* spp. In parallel, a chemical analysis by LC-MS/MS of the culture filtrate of the P strain revealed the presence of five acremeremophilane compounds, which were not detected in a Q strain analyzed earlier (PK Mukherjee, unpublished). We thus put forward a hypothesis that this unique PKS cluster may code for acremeremophilanes, which we proceeded to test by gene deletion experiment. Interestingly, in a transcriptome analysis, some members of this gene cluster were upregulated in a radiation-induced mutant (G2) of *T. virens* that produced larger amounts of secondary metabolites and downregulated in another radiation induced mutant (M7) that produced no detectable amounts of secondary metabolites (12, 13). We used the secondary metabolites overproducing mutant (G2) as a genetic tool for this study. Using gene knockout, we proved here that the PKS gene cluster is responsible for the biosynthesis of acremeremophilane metabolites ascertaining the polyketide origin of a large group of natural products that are being continuously discovered in fungi, especially from deep sea sediments and inside plants, and might be getting erroneously reported as sesquiterpenes.

## RESULTS

### The strain-specific PKS gene cluster.

**FIG 1:**
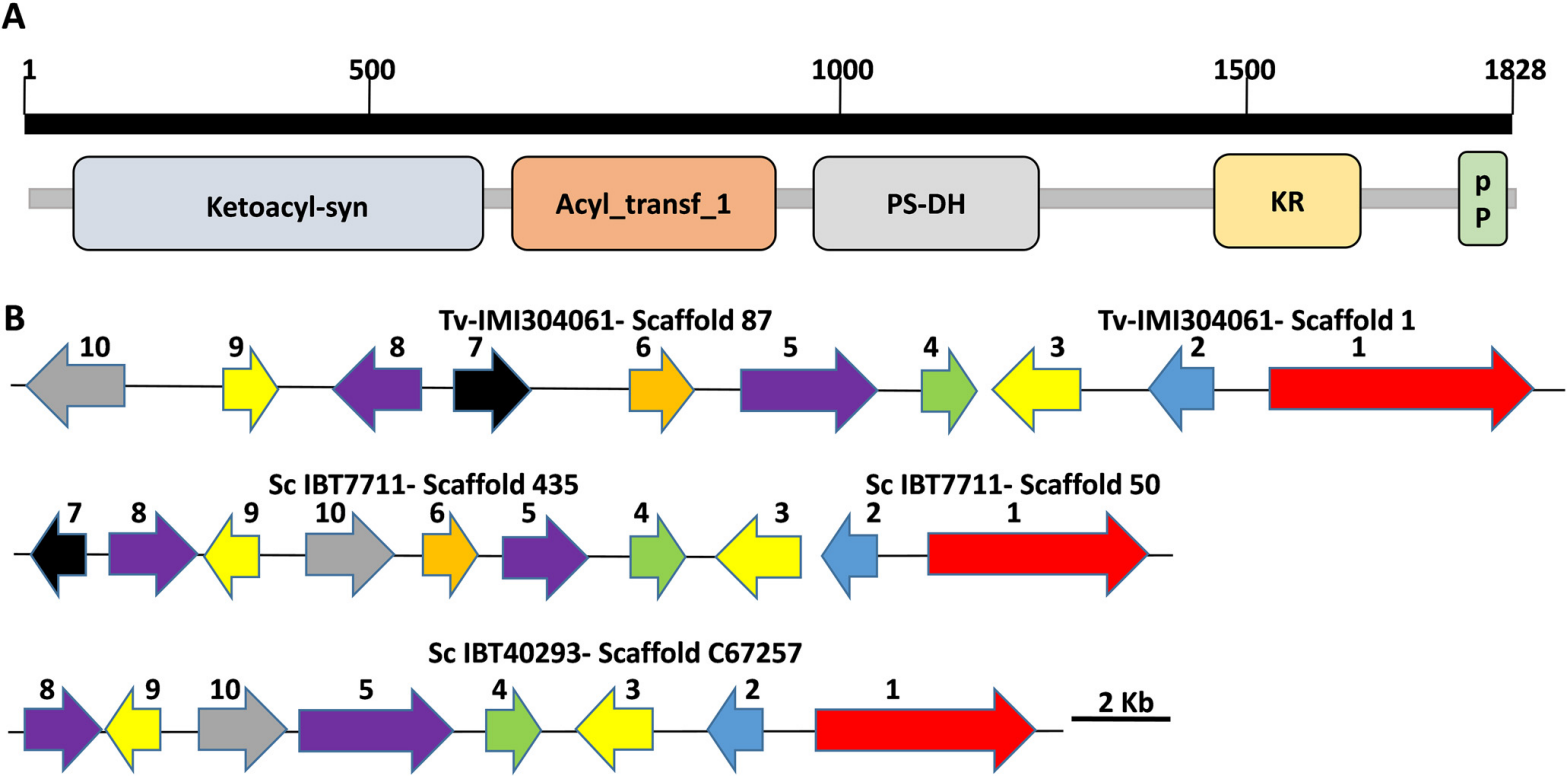
The polyketide synthase (PKS) and the gene cluster. (A) The domain organization of the *Trichoderma virens* highly reducing polyketide synthase AMM1. (B) The *amm* gene cluster in T. virens (Tv) IMI304061, Stachybotrys chartarum (Sc) IBT7711 and IBT40293. 1: Polyketide synthase, 2. Prenyl transferase, 3. FAD/FMN-containing dehydrogenase, 4. RmlC-like cupin, 5. Cytochrome P450, 6. Metallo-dependent hydrolase, 7. O-acyl transferase, 8. Cytochrome P450, 9. Rossmann fold NAD(P)+ binding dehydrogenase, 10. MFS transporter. PS-DH: Polyketide synthase-dehydratase; KR: Ketoreductase; PP: Phosphopantetheine attachment site.

Using a comparative genome analysis of the P versus Q genome, we identified a unique polyketide synthase gene in the P strain. Domain search by Pfam revealed it to be a highly reducing polyketide synthase (HRPKS) gene with a ketoreductase domain (Fig. 1A). The domain organization of this protein is similar to one HRPKS gene from *T. virens* Gv29-8 responsible for the biosynthesis of the salicylaldehyde metabolites (23). The gene cluster (designated *amm* cluster) is comprised of 9 other genes that include a prenyl transferase, two oxidoreductases, two cytochrome P450s, a hydrolase, an O-acyl transferase, and an MFS transporter (Fig. 1B). Intriguingly, the gene cluster is identical to a PKS gene cluster from Stachybotrys chartarum IBT 7711 and similar to the one from *S. chartarum* IBT 40293; the latter lacking *amm6* and *amm7* genes (Fig. 1B). All the genes (*amm1*- *amm10*) in *T. virens amm* cluster are highly homologous (≥70%) to the genes from *S. chartarum* (Table 1; Fig. S1). The *T. virens* cluster is distributed along two scaffolds (Scaffold 1 and 87), similar to that in *S. chartarum* IBT7711. However, the cluster in *S. chartarum* IBT 40293 is located on single scaffold.

**TABLE 1:** Orthology of the *amm* cluster genes

Gene no. in the cluster	cDNA no. in the assembly	Putative function/domain	Top BLAST hits Organism/Acc. No./% Identity/E value
*amm1*	CDS1812	Polyketide synthase	Stachybotrys chartarum IBT 40293/KFA45404.1/82.96/0.0 Stachybotrys chartarum IBT 7711/KEY74888.1/81.79/0.0
*amm2*	CDS1811	Prenyl transferase	Stachybotrys chartarum IBT 7711/KEY74889.1/84.47/0.0
*amm3*	CDS1810	FAD/FMN-containing dehydrogenase	Stachybotrys chartarum IBT 7711/KEY73630.1/71.4/0.0
*amm4*	CDS13310D	RmlC-like cupin	Stachybotrys chartarum IBT 40288/KFA74226.1/86.76/2e-174 Stachybotrys chartarum IBT 40293/KFA45405.1/80.51/3e-157 Stachybotrys chartarum IBT 7711/KEY73629.1/77.21/3e-149
*amm5*	CDS13310C	Cytochrome P450	Stachybotrys chartarum IBT 7711/KEY73628.1/84.02/0.0 Stachybotrys chartarum IBT 40293/KFA45406.1/83.24/0.0 Stachybotrys chartarum IBT 40288/KFA80927.1/84.30/0.0
*amm6*	CDS13310B	Metallo-dependent hydrolase	Stachybotrys chartarum IBT 7711/KEY73627.1/83.01/6e-174 Stachybotrys chartarum IBT 40288/KFA80926.1/82.03/1e-164
*amm7*	CDS13310A	O-acyl transferase	Stachybotrys chartarum IBT 7711/KEY73623.1/81.64/1e-118 Stachybotrys chartarum IBT 40293/KFA53562.1/81.22/1e-122
*amm8*	CDS13309	Cytochrome P450	Stachybotrys chartarum IBT 40288/KFA80928.1/91.59/0.0 Stachybotrys chartarum IBT 40293/KFA45408.1/90.64/0.0 Stachybotrys chartarum IBT 7711/KEY73624.1/88.57/0.0
*amm9*	CDS13308	Rossmann fold NAD(P)+ binding dehydrogenase	Stachybotrys chartarum IBT 7711/KEY73625.1/69.68/1e-150 Stachybotrys chartarum IBT 40293/KFA45409.1/63.37/9e-126
*amm10*	CDS13307	MFS transporter	Stachybotrys chartarum IBT 40293/KFA45407.1/72.84/0.0 Stachybotrys chartarum IBT 40288/KFA80926.1/72.84/0.0 Stachybotrys chartarum IBT 7711/KEY73626.1/68.71/0.0

### Deletion of the PKS gene abolishes biosynthesis of acremeremophilanes.

**FIG 2:**
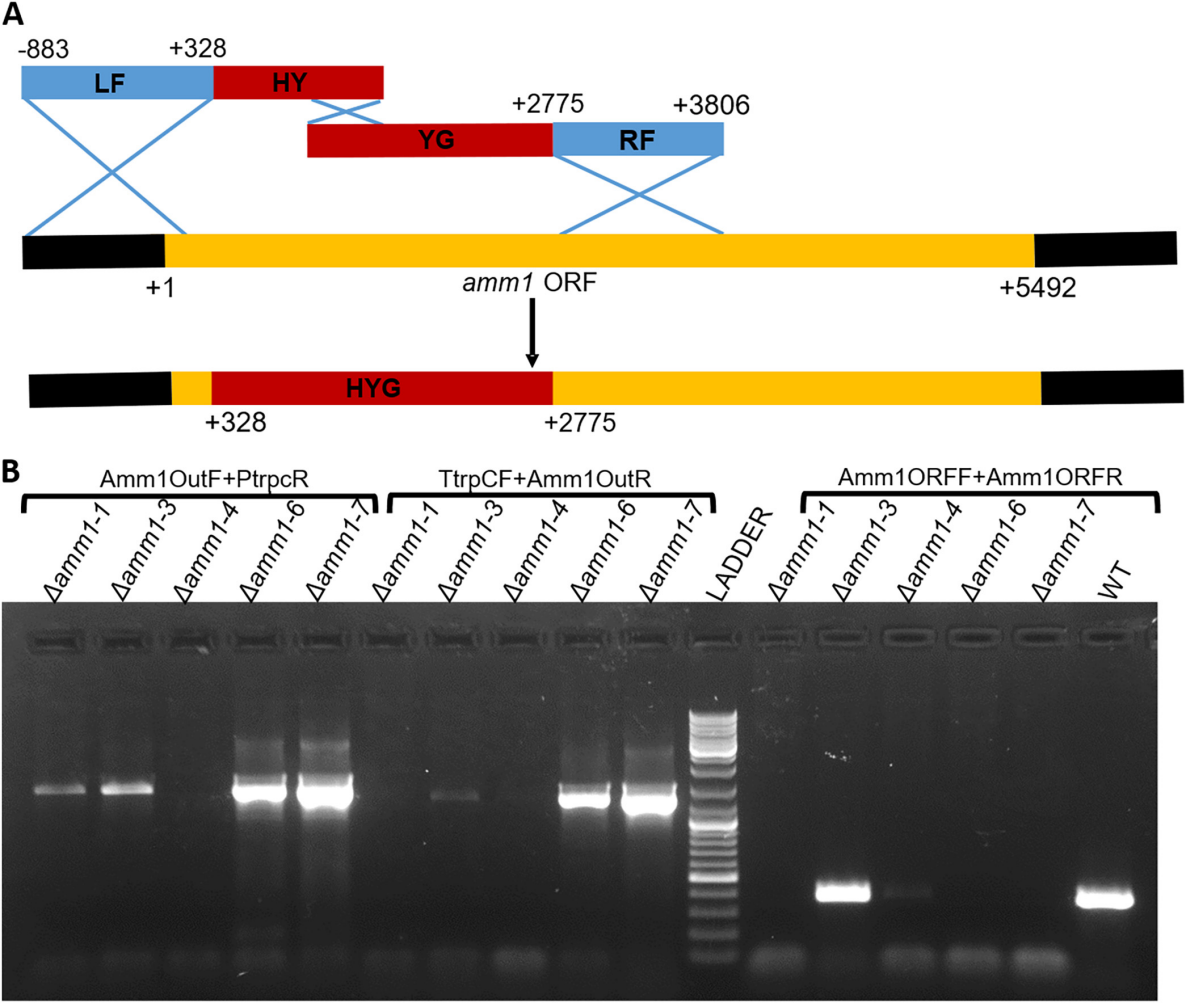
Strategy for gene deletion in *Trichoderma virens* using homologous recombination (A) and Confirmation of homologous recombination and gene deletion in *amm1* knockout mutants (B). Transformant numbers 6 and 7 were selected for further analysis.

**FIG 3:**
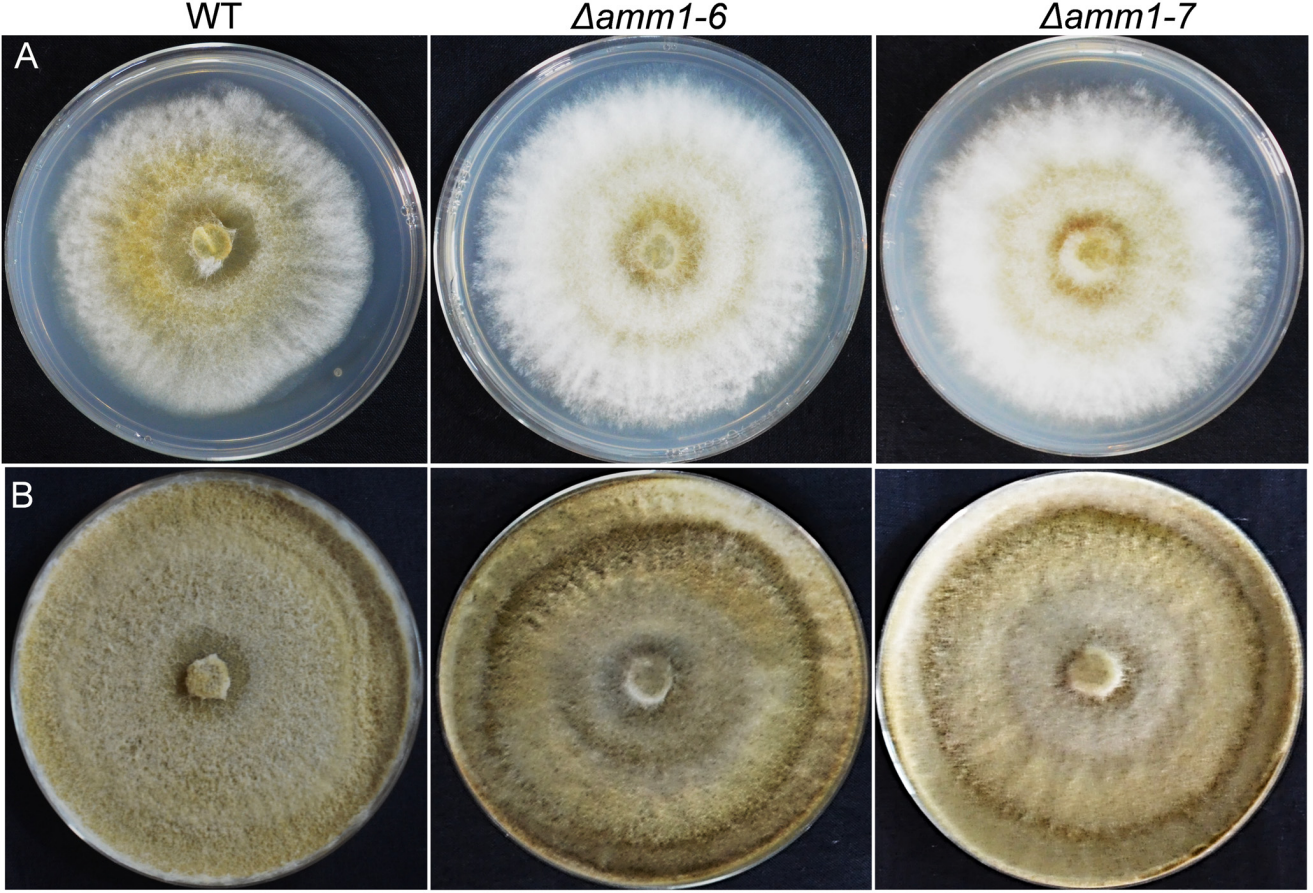
Growth characteristics of *Trichoderma virens* G2 and two independent knockout mutants. (A) 2 days after inoculation and (B) 7 days after inoculation.

**FIG 4:**
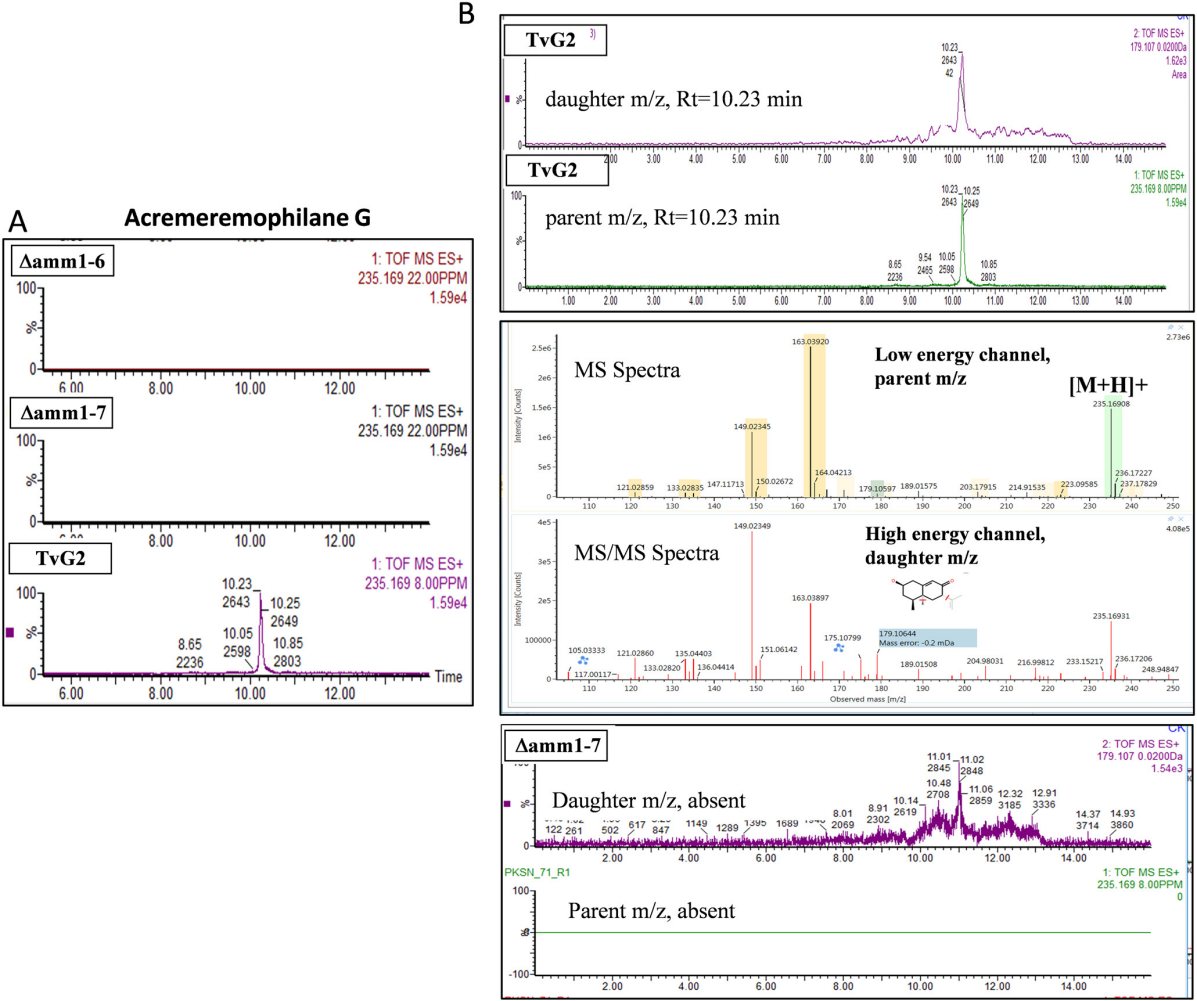
Detection of Acremeremophilane G. (A) Overlaid extracted ion chromatograms depicting the detection of Acremeremophilane G in *Trichoderma virens* G2 and its absence in knockout mutants, and (B) Spectral identification to confirm the detection using high resolution accurate mass analysis.

Using split-marker based homologous recombination (Fig. 2), we obtained five stable putative mutants which were screened for locus-specific homologous recombinants using primer pairs Amm1OutF and PTrpCR (one upstream of the left flank and other from the hygromycin resistance cassette, hygR) and TTrpCF and Amm1OutR (one from hygR cassette and another from downstream of the right flank), as well as presence or absence of the wild-type gene by gene specific primers (Amm1ORFF and Amm1ORFR). Based on the stability and absence of wild type allele, we finally selected two gene deletion mutants (Δ*amm1-6* and Δ*amm1-7*) (Fig. 2B). On agar medium, the mutants were fast growing compared to the parental strain G2, with about 18% higher radial growth (Fig. 3). We analyzed the metabolite extract by high resolution accurate mass analysis. The acremeremophilanes G, I, K, N, and O were detected, identified and confirmed in G2 while they were found to be absent in the two independent mutants (Fig. 4, Fig. S2-S5).

### Proposed biosynthesis pathway.

**FIG 5:**
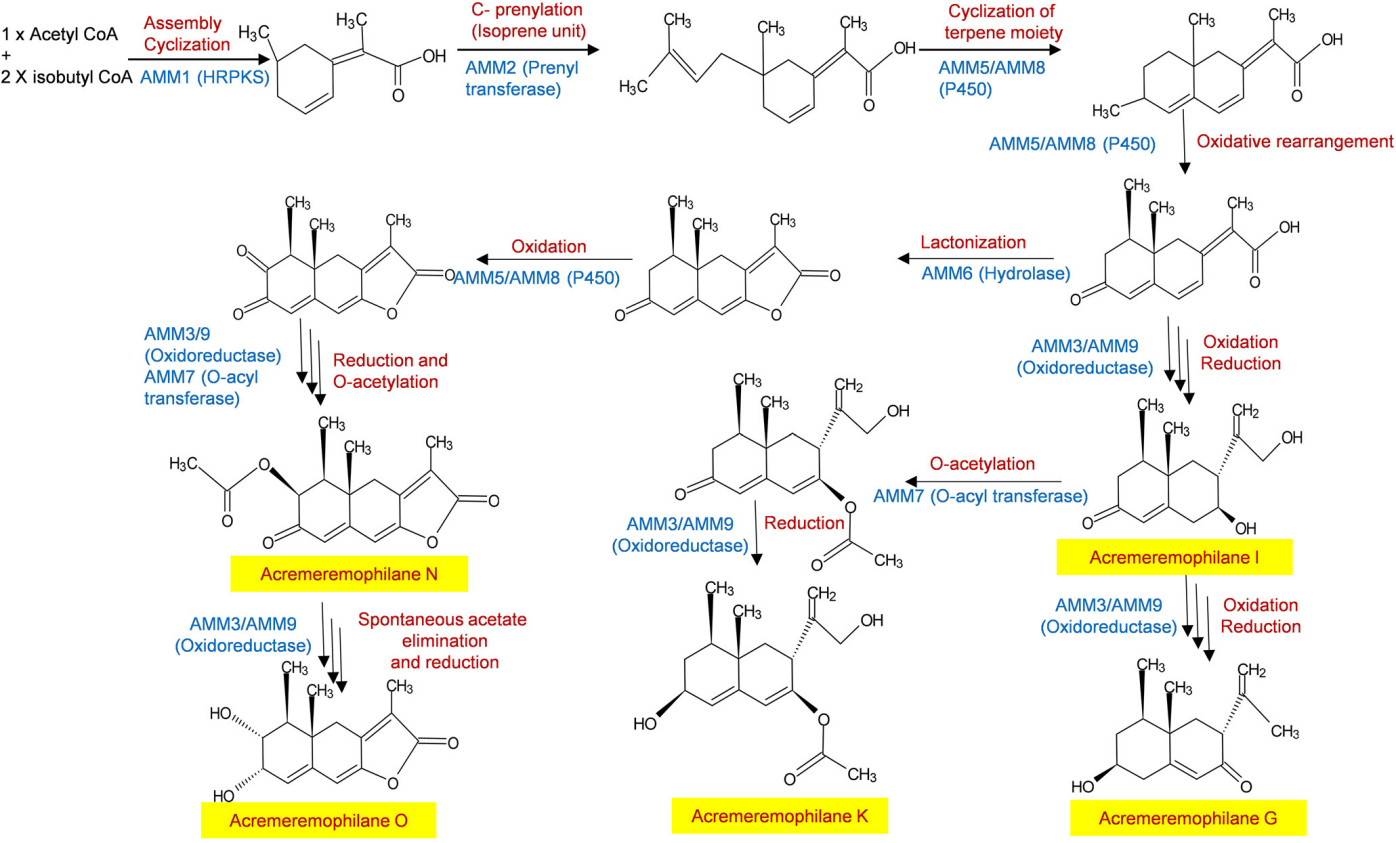
The proposed biosynthesis pathway of acremeremophilanes, catalyzed by the enzymes coded by the genes present in the *amm* gene cluster. The highlighted compounds were detected in *Trichoderma virens* G2 and not in the *amm1* deletion mutants.

Taking cue from Matsuda et al. (24) on biosynthesis of the meroterpene anditomin in Aspergillus *variecolor*, we propose a novel pathway for the biosynthesis of acremeremophilane metabolites (Fig. 5). The HRPKS enzyme assembles the 10-carbon compound by using one molecule of acetyl-CoA and two molecules of isobutyryl CoA, which is reduced and cyclized by the same enzyme. The attachment of an isoprene unit is accomplished by AMM2, the prenyl transferase located next to the PKS gene in the cluster. The cyclization of the terpene moiety is proposed to be performed by one of the two cytochrome P450s (AMM5 or AMM8), leading to the bicyclic scaffold which undergoes oxidative rearrangements to provide a common intermediate for both the bicyclic and tricyclic products. This precursor undergoes oxidation/reduction to produce acremeremophilane I and G. Acetylation (accomplished by an O-acyl transferase located in the cluster) of acremeremophilane I, followed by a reductive reaction leads to acremeremophilane K. Lactonization, accomplished by the hydrolase (AMM6) leads to biosynthesis of precursor for tricyclic compounds. Oxidation/reduction and acetylation results in the formation of acremeremophilane N, which undergoes acetate removal and reduction to form acremeremophilane O. Once produced, these metabolites are transported outside the cell by the MFS transporter AMM10, present in the same biosynthesis gene cluster.

## DISCUSSION

Microbial natural products have immense impact in human welfare, especially in the health and agriculture sectors. Correct classification of such products is of utmost importance as this information is required for ease of chemical synthesis and discovery of pathways and enzymes involved, which is again important if one has to perform experiments for rational drug designing, pathway engineering, and overexpression of genes for such metabolites.

Eremophilanes, discovered nearly 90 years ago, are a large group of clinically significant natural products mainly produced by plants and fungi (25). The eremophilanes are described as sesquiterpenes, synthesized by cyclization of farnesyl pyrophosphate by terpene cyclases. Our study shows that a group of these metabolites have been wrongfully classified, perhaps due to the absence of information on genetics and biochemical pathways. Using a radiation-induced, secondary metabolite overproducing mutant, and gene knockout, backed by strong bioinformatics and genome mining, coupled with high resolution accurate mass analysis, we provide evidence that the tricyclic eremophilanes like acremeremophilane I, G, K, N, and O are synthesized by a highly reducing polyketide synthase, and not by a terpene cyclase. This HRPKS gene cluster also has a prenyl transferase, and the cluster is absent in Gv29-8 genome.

The basic 10-carbon skeleton is somewhat similar to 3,5–dimethylorsellinic acid (DMOA), a precursor for anditomin biosynthesis in Aspergillus *variecolor* (24). Anditomin is a meroterpene which is produced by fusion of a farnesyl moiety to DMOA, followed by other modifications (in case of acremeremophilanes, an isoprene unit is added to the polyketide skeleton- this study). Several genes in the *amm* cluster and the *and* cluster are also functionally similar. However, the PKS in *and* cluster is a nonreducing one while in the *amm* cluster, it is a highly reducing PKS. The cyclization of the terpene moiety is catalyzed by a terpene cyclase in case of anditomin biosynthesis, while in case of acremeremophilanes biosynthesis in *T*. *virens*, this is most likely accomplished by a cytochrome P450, similar to viridicatumtoxin biosynthesis in *Penicillium aethiopium* as described by Chooi et al. (26). Oxidative rearrangements are accomplished by oxidoreductases and lactonization by hydrolases, in case of both, the anditomin and acremeremophilanes biosynthesis. A major difference is the use of precursors- in case of anditomin, it’s malonyl CoA, while it’s isobutyryl CoA in case of acremeremophilanes (both these pathways use acetyl-CoA as a starter unit). Isobutyryl CoA as the substrates for PKS is known for other metabolites (27, 28).

First reported in 1932 by Bradfield et al. (29), eremophilane “sesquiterpenes” continue to be a major class of plant and fungal natural products, with more than 180 compounds discovered in fungi alone (25, 30). Many of these are bioactive and hence have attracted attention as antimicrobial, anticancer, immunomodulatory, and phytotoxic compounds. Despite their abundance, the genetic basis and biosynthesis routes for most of such compounds are unknown. It is documented that the 15-carbon bicyclic eremophilane backbone is a sesquiterpene (25). Tricyclic acremeremophilane-like metabolites are also abundant in nature. For example, berkleasmins C,D,E from *Berkleasmius nigroapicale* (31), acremeremophilanes L,M,O from *Acremonium* sp. (32), eutymeremophilane A from *Eutypella* sp. (33), rhizoperemophilanes K and L from *Rhizopycnis vagum* (31), and similar metabolites from *Xylaria* sp. (34) and *Glomerella cingulata* (35). However, the genetics and biosynthesis route for the tricyclic eremophilanes have not been worked out even though it was hypothesized that such compounds are derived from eremophilane backbone (31). Due to this reason, these metabolites, being continuously discovered in fungi from deep sea sediments and endophytic fungi, are being reported as sesquiterpenes, which, according to our findings, should be described as meroterpenes (hybrid of polyketide and terpene).

The present finding is important on several counts: (i) This is the first report of existence of eremophilane compounds in the plant beneficial fungus *Trichoderma.* (ii) The compounds are synthesized only by P strain of *T. virens* encoded by a strain-specific gene cluster. (iii) Identical gene cluster is present in the toxic mold *S. chartarum*, but absent in other *Trichoderma* spp., including a Q strain of *T. virens*. (iv) A biosynthesis pathway is proposed for the polyketide route of biosynthesis for these compounds described in literature as sesquiterpenes, necessitating the reclassification of a large number of natural products.

It would be interesting to see what metabolite is coded by the same cluster in *S. chartarum*, as the genome is rich in secondary metabolism gene clusters and the mold produces several meroterpenes (36, 37).

### Conclusion.

Using a combination of bioinformatics, genome analysis, gene deletion experiments, and high-resolution accurate mass analysis, we provide evidence that the acremeremophilanes in *T. virens* are synthesized via a polyketide route, and not by terpene cyclase, as is widely believed. These metabolites thus need to be reclassified as meroterpenes and not sesquiterpenes. The current findings would have implications in the discovery and deployment of these medicinally important compounds.

## MATERIALS AND METHODS

### Fungal strain and growth conditions.

We used a gamma ray induced mutant (G2) of *T. virens* for gene deletion experiment, as this strain overproduces secondary metabolites and also is upregulated in some of the genes present in the PKS gene cluster under study (12). The fungus was cultivated routinely on potato dextrose medium (PDB or PDA, when agar was used) or Vogel’s minimal salt medium with 1.5% glucose (VMG). The strain was maintained at −80C for long term storage.

### Identification of the gene cluster and its homology.

The gene cluster was identified by comparison of the genome of two strains of *T. virens*- the Q strain Gv29-8 (https://mycocosm.jgi.doe.gov/TriviGv29_8_2/TriviGv29_8_2.info.html; 22) and the P strain IMI 304061 (accession number LQCH01000000; 19). The *Stachybotrys* cluster was identified by homology search from the sequences available in the database (36).

### Gene deletion experiment and characterization of the mutants.

For deletion of the PKS gene in *T. virens* G2, a modified split marker protocol was followed (16). Briefly, the 5′ flank was amplified with the primer pair Amm1FKpnI (all oligonucleotide primer sequences are listed in Table S1) and Amm1RXhoI with KpnI and XhoI enzyme sites integrated in the sequence, and the 3′ flank was amplified with the primer pair Amm1FXbaI and Amm1FSacI with XbaI and SacI enzyme sites integrated. The flanks generated were ligated to pATBS (a plasmid harboring the hygromycin resistance cassette) linearized with the respective enzyme pairs. This placed the left flank above the hyg cassette and right flank under the hyg cassette in two separate plasmids. The flanks with part of the hyg cassette were generated with Amm1FKpnI and NLC37 for left flank and NLC38 and Amm1RSacI for right flank. Protoplasts were generated from *T. virens* G2 germinated conidia and transformed with these two constructs added in equimolar ratio (16). The plates were incubated for 1 day and overlaid with 1% water-agar amended with 600 ppm hygromycin B (final concentration 200 ppm). The colonies that appeared two-3 days after the hyg overlay were picked-up, transferred to fresh PDA plates with 100 ppm hyg, purified three times by single spore isolation and then subjected to molecular analysis to select the gene deletion mutants based on the absence of the wild type band. The deletion mutants were phenotyped for growth characteristics and production of metabolites. All the experiments were performed in three replicates and repeated at least twice.

### Extraction and analysis of the filtrates.

*T. virens* G2 and the knockout mutants were grown in VMG medium with shaking at 28 C for 4 days before solvent extraction of the metabolites (Bansal et al. 2021). After drying *in vacuo*, the samples were reconstituted in methanol:water (1:1) and analyzed by high resolution LC-MS/MS as described before (21). Briefly, the analysis was performed in an Acquity Ultra Performance Liquid Chromatograph (UPLC), coupled with a QToF-MS (Synapt G2 HDMS, Waters Corporation, Manchester, UK). Ionization was performed with electrospray ionization (ESI) in positive polarity at the mass resolution of 20,000. MassLynx 4.1 software was used to control the QToF and the data were acquired in full scan MSe mode at low energy at 4 V followed by high energy ramping at 10 to 60 V. For chromatographic separation of metabolites, an Acquity UPLC HSS T3 column (2.1 × 100 mm, 1.8 μm, Waters India Pvt. Ltd., Bengaluru) was used, with the following source parameters: capillary 1 kV, sampling cone 10 V, source temperature 120°C, desolvation temperature 500°C, desolvation gas flow 1000 L/h, and cone gas flow 50 L/h. Mobile phase A was methanol:water (10:90, vol/vol) and phase B was methanol:water (90:10, vol/vol) with 0.1% formic acid, in both phases. The analysis was performed in three replicates and repeated twice.
