# Expression of Concern for Bansal et al., ‘Genetic Evidence in Favor of a Polyketide Origin of Acremeremophilanes, the Fungal “Sesquiterpene” Metabolites’

**DOI:** 10.1128/spectrum.03664-23

**Published:** 2023-11-17

**Authors:** American Society for Microbiology

## EXPRESSION OF CONCERN

The American Society for Microbiology (ASM) and *Microbiology Spectrum* are issuing this Expression of Concern regarding the following publication:

Bansal R, Sethy SK, Khan Z, Shaikh N, Banerjee K, Mukherjee PK. 2022. Genetic evidence in favor of a polyketide origin of acremeremophilanes, the fungal “sesquiterpene” metabolites. Microbiol Spectr 10:e01793-22. https://doi.org/10.1128/spectrum.01793-22.

It was brought to the attention of ASM and *Microbiology Spectrum* that the model presented in Fig. 5 of this research article may not be chemically plausible, which further raises concerns with core conclusions and the title of the article. The main issues raised are as follows:

1. The class of fungal secondary metabolites called acremeremophilanes, which are generally regarded to be typical sesquiterpenes, are proposed to be meroterpenoids in this paper, but this proposal is potentially not fully supported by the data presented in genetic experiments. Therefore, this primary conclusion of the paper, also reflected in the title of the article, may not be supported.

1. The proposed reactions in Fig. 5 are likely chemically implausible, which brings into question the proposed pathway itself. Specifically, the concerns mention the example of isobutyl-CoA, which cannot be used as an extender unit because it does not contain the required second carboxy-group. Per definition, a polyketide synthase condenses short chain carboxylic acids with activated dicarboxylic acids in a condensation reaction.

This Expression of Concern is issued to raise awareness of the readers to above-mentioned concerns.

