# Supplementary material for: Genetic Evidence in Favor of a Polyketide Origin of Acremeremophilanes, the Fungal “Sesquiterpene” Metabolites
Source: Microbiol Spectr. 2022 Aug 8;10(4):e01793-22. doi: 10.1128/spectrum.01793-22 (PMC9430172; doi:10.1128/spectrum.01793-22)

Suppl. Information

Genetic evidence in favour of a polyketide origin of acremeremophilanes, the fungal "sesquiterpene" metabolites

Table S1. Oligonucleotide primers used in this study

Primer Name	Sequence (5'-3')	Purpose
Amm1FKpn1	TTTGGTACC TCTGAACCGAGA AAAGGAC	Amplification of 5' flank of *amm1* gene
Amm1RXho1	AAACTC GAG GTAGTCCGT GTTAGT	
Amm1FXba1	TTTTCTAGAACAACACGCTGCTAG GC	Amplification of 3' flank of *amm1* gene
Amm1RSac1	AAAGAGCTCCTTGACGTCGCT	
Amm1ORFF	CCGTCTCTCACGCTATCCTC	Confirmation of gene deletion
Amm1ORFR	ACCGGAGAAAATCCACACTG	
Amm1OutF	GTGGATTCGAATGGGCTG ATC	Confirmation homologous recombination
Amm1OutR	GGCGGA GTC ATA GTCGTC	Confirmation of homologous recombination
PtrpCR	TACAAGTGGGCTGATCTGACC	Confirmation of homologous recombination
TtrpcF	CCTACAGGACACACATTCATCG	Confirmation of homologous recombination
NLC37	GGATGCCTCCCCTCGAAGTA	Primer from hyg cassette
NLC38	CGTTGCAAGACCTGCCTGAA	

Figure S1. Alignment of *Trichoderma virens* sequences with *Stachybotrys* orthologues

Amm1

```
amm1:CDS1812        -MASHEDTAPPSISPSLEMFTPQVHSAASIDTGVSCDNCTSDGEAPQASDTDIAIIGMAC  59
Sch:KFA45404.1       MVSIHDDTAPPSASLSPQVFTPVYSE-ASVDTSV-----DHDSAAPQANDDDIAIIGMAC  54
Sch:KEY74888.1      MVSIHDDTAPPSASLSPQVFTPVYS---SVDTSV-----DHDSAAPQANDDDIAIIGMAC  52
                     :: *:****** * * ::***      *:**.*       *. ****.* *********

amm1:CDS1812        RVPGSNNSPAELWQYLLDKGDASGSMPAMRWEPYNHRHPRNAAVLTRTTSTGYFLDRIED  119
Sch:FA45404.1       RVPGSNNSPAELWQYLLNKGDASGTMPAMRWEPYNHRNPKNAGILSKTTSTGYFLDRIED  114
Sch:KEY74888.1      RVPGSNNSPAELWQYLLNKGDASGTMPAMRWEPYNHRNPKNAGILSKTTSTGYFLDRIED  112
                    *****************:******:************:*:**.:*::*************

amm1:CDS1812        FDASFFAVSPREAEQIDPQQRITLEVAWEALEDAGIPADTLSGSDTAVFMGVNSDDYGKL  179
Sch:FA45404.1       FDASFFAVSPRETEQIDPQQRITLEVAWEALEDAGIPADSLSGSDTSVYMGVNSDDYGKL  174
Sch:KEY74888.1      FDASFFAVSPREAEQIDPQQRITLEVAWEALEDAGIPADSLFGSDTSVYMGVNSDDYGKL  172
                    ************:**************************:* ****:*:**********

amm1:CDS1812        VLEDLEGVGAHMGVGTAYCGIPSRVSYHLDLLGPSVAVDAACASSLVAVHQARAALLAGE  239
Sch:FA45404.1       VLEDLEGVGAHMGVGTAYCGIPSRVSYHLDLFGPSVAVDAACASSLVAVHQARQAILAGE  234
Sch:KEY74888.1      VLEDLEGVGAHMGVGTAYCGIPSRVSYHLDLFGPSVAVDAACASSLVAVHQARQAILAGE  232
                    *******************************:********************* *:****

amm1:CDS1812        TKLALAGGVNALIGPGLTRVLDEAGAIASDGKCRSFDDSAHGYGRGEGAGVVVLKKLTKA  299
Sch:FA45404.1       TKLAIAGGVNALIGPGLTRVLDEAGTIASDGKCRSFDESAHGYGRGEGAGVVVLKKLSKA  294
Sch:KEY74888.1      TKLAIAGGVNALIGPGLTRVLDEAGAIASDGKCRSFDESAHGYGRGEGAGVVVLKKLSKA  292
                    ****:********************:***********:*******************:**

amm1:CDS1812        LADGDRVHAVLKGSAVAADGRTLGIMAPNPAAQQLVAQKALQEARTPAETISYIEAHATS  359
Sch:FA45404.1       LVDGDRVHAVLKGSAVAADGRTVGIMAPNSAAQQLVAVKALQDARTPAETISYIEAHATL  354
Sch:KEY74888.1      LVDGDRVHAVFKGSAVAADGRTVGIMAPNSAAQQLVAVKALQDARTPAETISYIEAHATS  352
                    *.********:***********:****** ******* ****:****************

amm1:CDS1812        TPLGDPTEMAALAKVYGQGARQGPEGGSSCQVGSIKSNIGHLEAAAGVMGLIKAAMVLKH  419
Sch:FA45404.1       TPLGDPTEMAGLAKVYGEGAP----RLSSCQVGSIKSNIGHLEAAAGVMGLIKAAMVLKH  410
Sch:KEY74888.1      TPLGDPTEMAGLAKVYGEGAP----RLSSCQVGSIKSNIGHLEAAAGVMGLIKAAMVLKH  408
                    **********.******:**       *********************************

amm1:CDS1812        GLVPGQANLETLSTKIDWANNGMTVSKDTTRLVYSPFTPRRAAVASYGYSGTVSHAILEA  479
Sch:FA45404.1       GVVPGQANLETLSSKIDWTNNRMIVNKDARQLAGSPVNPRRAAVASYGYSGTVSHAVLEA  470
Sch:KEY74888.1      GVVPGQANLETLSSKIDWTNNRMIVNKDARQLAGSPVNPRRAAVASYGYSGTVSHAVLEA  468
                    *:***********:****:** * *.**: :*. **..*****************:***

amm1:CDS1812        SPEATTPLAVVGMSEEADNQPALLVISGPQESRIPKAAAVLADWLASSAGAAAPLHHVAN  539
Sch:FA45404.1       SPEAALSVAV----NTPENVAALLFLSGPQESRIPKAAAALADWLASPAGAAAPLHHVAN  526
Sch:KEY74888.1      SPEAALSVAV----NTPENVAALLFLSGPQESRIPKAAAALADWLASPAGAAAPLHHVAN  524
                    ****:  :**     :  :*  ***.:************.******* ***********

amm1:CDS1812        TLARHRSHHRFRIAITATDKDEAISLLQKVSNGQTDKLITSGRVSPNKPNNTGGAVWIFS  599
Sch:FA45404.1       TLARHRSHHRFRTAITATTKQEAVSLLQRVASGQSNKLITSGRVGT----NTAGPVWIFS  582
Sch:KEY74888.1      TLARHRSHHRFRTAITATTKQEAVSLLQRVASGQSNKLITSGCVGT----NTAGPVWIFS  580
                    ************ ***** *:**:****:*:.**::****** *.     **.* *****

amm1:CDS1812        GHGAQWPAMGQELLATQPAFAQTIQQLEPIVQAELDGFSVSKALEKGEESWTSDEIQVLT  659
Sch:FA45404.1       GHGAQWPTMGQELMATQPAFARTIQKLEPVVQAELDGFSVSKALEKGEESWTSDEIQVLT  642
Sch:KEY74888.1      GHGAQWPTMGQELMATQPAFARTIQKLEPVVQAELDGFSVSKALEKGEESWTSDEIQVLT  640
                    *******:*****:*******:***:***:*****************************

amm1:CDS1812        FAMHVGLASVLTETAGPPSAIVGHSLGETAAAVVAGSISLEDGARLVARRAKLYRRHMGK  719
Sch:FA45404.1       FAMHVGLANVLCETAGPPSAIVGHSLGETAAAVVAGAIDLEDGAKLVSRRARLYRRHMGK  702
Sch:KEY74888.1      FAMHVGLANVLCETAGPPSAIVGHSLGETAAAVVAGAIDLEDGAKLVSRRARLYRRHMGK  700
                    ********.** ************************:*.*****:**:***:*******

amm1:CDS1812        GAMALVSLSIDEVRKKLDASTEYSDIDIAIDASPTSCVVGGPSESVTKLSEQWENEGVQV  779
Sch:FA45404.1       GAMALVSLGVDEVRKKLNASPEYGDIDIAIDASSTSCVLAGTSELVTKLSEQWKSEDIQV  762
Sch:KEY74888.1      GAMALVSLGVDEVRKKLNASPEYGDIDIAIDASSTSCVLAGTSELVTKLSEQWKSEDIQV  760
                    ********.:*******:** **.********* ****:.* ** ********:.*.:**

amm1:CDS1812        RAVKSDMAFHSRMVHDLMDPLRQYLSGFCSWTPKIKLYSTSAQDARSTGPRDVEYWVNNM  839
Sch:FA45404.1       PAVKSDMAFHSRMLLDLMDPLRRCLPDFRSWTPKIKLYSTSAQDARFTGPRDVEYWVNNM  822
Sch:KEY74888.1      PAVKSDMAFHSRMLLDLMDPLRRCLPDFRSWTPKIKLYSTSAQDARFTGPRDVEYWVNNM  820
                     ************: *******: * .* **************** ************
```

```
amm1:CDS1812        VAPVRLREATQALAEDGYRTFVEVSAHPIVVHSVSETLEAMDLGSDVLVLPTMVRNKAVM   899
Sch:FA45404.1       VAPVRLREATQALAQDGHRMFVEVSAHPIVADSVSETLEAMDLGSDVLVLPTMLRNKPVI   882
Sch:KEY74888.1      VAPVRLREATQALAQDGHRMFVEVSAHPIVADSVSETLEAMDLGSDVLVLPTMLRNKPVI   880
                    **************:**:* **********..*********************:*** *:

amm1:CDS1812        ERVLTTVGSLHVSGQKTAYTSHNAKRPWLP-DVPRTIWNHEPYWRTVAKVPLNEVTSHKP   958
Sch:FA45404.1       ERILTTVGSLHVSGQKTAYTSHNAKRPWLSEGVPRTIWNHEPYWRTVAKVPLNEVTAHKP   942
Sch:KEY74888.1      ERILTTVGSLHVSGQKTAYTSDNAKRPWLSEGVPRTIWNHEPYWRTVAKVPLNEVTAHKP   940
                    **:******************.*******  .************************:***

amm1:CDS1812        ENNTLLGTRTAIWGTGNVLYQTTLDEESRPYPGRHPLHGSEIVPAAVLINTFLHAIDTET   1018
Sch:FA45404.1       ENNTLLGTRTDIWGTRNVLYQTQLDEESRPYPGRHPLHGSEIVPAAVLINTFLHAIDTET   1002
Sch:KEY74888.1      ENNTLLGTRTDIWGTRNVLYQTQLDEESRPYPGRHPLHGSEIVPAAVLINTFLHAIDTET   1000
                    ********** **** ****** *************************************

amm1:CDS1812        SCLEGMSLKVPVMITPPRQIQILVSNQQQISLTSRLADSGGGTWLVNTTSRIMPAAGDAR   1078
Sch:FA45404.1       NCLESLSLKVPVIISPPRQIQILVSDQQQISLTSRLAESG-GAWLVNTTGRVISTVGDAR   1061
Sch:KEY74888.1      NCLESLSLKVPVIISPPRQIQILVSDQQQISLTSRLAESG-GAWLVNTTGRVISTVGDAG   1059
                    .***.:******:*:**********:***********:** *:******.*:: :.***

amm1:CDS1812        PERLDLGKLQTRLQDKLAETFSIDYLAKVGVAEMGFPWAVTSHRALGDNEFLACVETNPN   1138
Sch:FA45404.1       PERLDLEKLQTRLPEKLAETFSIDYLDKVGVPEMGFPWAVTSHRALGDDEFLACVETNPN   1121
Sch:KEY74888.1      PERLDLEKLQTRLPEKLAETFSIDYLDKVGVPEMGFPWAVTSHRALGDDEFLACVETNPN   1119
                    ****** ****** :*********** **** ****************:***********

amm1:CDS1812        KVDCAGLNDLGASIMDAATSIASTIFFKDPLLRMPTAVDRIMKYGQLDTLQEGWIYCKRS   1198
Sch:FA45404.1       QAESQGLNDLGASIMDAATSIASTIFFKDPLLRMPTAVDRIIKYGQLETLAQGWIHCKRS   1181
Sch:KEY74888.1      QAESQGLNDLGASIMDAATSIASTIFFKDPLLRMPTAVDRIIKYGQLETLAQGWIHCKRS   1179
                    :.:. ************************************:*****:** :***:****

amm1:CDS1812        QDKSKPFAVDVLVSDTAGTVLVEFAAMAFAGLENDAVSRKSTRGLVHKLAWPPAKLVETP   1258
Sch:FA45404.1       QDKSKPFAVDVVVSDAAGNALIEFVAMTFAGLENDAVSRKSTRGLVHRLAWPPAKMAETP   1241
Sch:KEY74888.1      QDRSKPF----------------------AGLENDAVSHKSTRGLVHRLAWPPAKMAETP   1217
                    **:****                      *********:********:*******:.***

amm1:CDS1812        TTFRHVVLFSTEHDGKEQESHRRYTHQLTSRGYTVSVASDVKELASLGADTIVVDLAHSS   1318
Sch:FA45404.1       INLNHVVLFSTENDGRETEAKCQYTRQLIKRGHTISTTRDVKELASLGDDTIVLDLAHSS   1301
Sch:KEY74888.1      INLNHVVLFSTEHDGRETEAKCRYTRQLIKRGHTVSTARDVKELASLGDDTIVLDLAHSS   1277
                     .:.********:**:* *:: :**:** .**:*:*.: ********* ****:******

amm1:CDS1812        DDYDSALDAASAACASLISAVQTMAASPDCKATLFSLVPHHCQDDAEEIDLASAPLYGLA   1378
Sch:FA45404.1       GSYASALDAASASCNSLVSAAQAMAASPDCKAKLFSIVPQHHQAEAEDIHLASASLYGLA   1361
Sch:KEY74888.1      GSYASALDAASASCNSLVSAAQAMAASPDCKAKLFSIVPQHHQAEAEDIDLASASLYGLA   1337
                    ..* ********:* **:**.*:*********.***:**:* * :**:*.**** *****

amm1:CDS1812        RIIKSEHPDLFAGLFQTTADGNLPLAAMRYVHGENIVKQDVDGVARVARLRPFSADRKDG   1438
Sch:FA45404.1       RIIKSEHPDLFGGLFEM-YDGKLPLAAIRYVQGENIVKQDDDGVARVGRLWPFVADGKDA   1420
Sch:KEY74888.1      RIIKSEHPDLFGGLFET-YDGKLPLAAIRYVQGENIVKQDDDGVARVGRLRPFVADGKDA   1396
                    ***********.***:   **:*****:***:******** ******.** ** ** **.

amm1:CDS1812        DQAPRQLRFSSAGTYLITGGLGALGLVVAKWMVDRGARRLLLVSRRELPCRTQWADIIDS   1498
Sch:FA45404.1       DQAPKQLRFSPAGTYLITGGLGALGLVVAKWMVERGARRLLLVSRRSLPLSYSLQPTQAR   1480
Sch:KEY74888.1      DQAPKQLRFSPAGT----------------------------------------------   1410
                    ****:***** ***

amm1:CDS1812        SPYYGIIDAILGMEAAGASVHVLPVDISAPNASTSLGSAIHSLSLPRVTGVIHAAGVLVD   1558
Sch:FA45404.1       KS----------------------------------------------------------   1482
Sch:KEY74888.1      ------------------------------------------------------------   1410

amm1:CDS1812        QMVQEVDRASFDAVLAPKLNGALALDELFPPGTLDFFTLFSSCGQLLGFPGQASYAAANA   1618
Sch:FA45404.1       ------------------------------------------------------------   1482
Sch:KEY74888.1      ------------------------------------------------------------   1410

amm1:CDS1812        CLDAIAQRRRARGDKGAHSILWTSWRGMGMAASTEYINAELEARGISDVTAEEAFLAWDC   1678
Sch:FA45404.1       ------------------------------------------------------------   1482
Sch:KEY74888.1      ------------------------------------------------------------   1410

amm1:CDS1812        IASADTPEGDGLAVVLRALPIDHDEEVAHPILEDIAPRKAPPAAAADVAGDGTASGTANK   1738
Sch:FA45404.1       ------------------------------------------------------------   1482
Sch:KEY74888.1      ------------------------------------------------------------   1410
```

```
amm1:CDS1812        GAAQGRPASGPELESWLDEQLKGAVADTLGMATADVDAAVALSEMGMDSVLTVSFRTRVQ   1798
Sch:FA45404.1       ------------------------------------------------------------   1482
Sch:KEY74888.1      ------------------------------------------------------------   1410

amm1:CDS1812        KMLKVKMGPTLAWKCPTVGHLVKHFASEIQ   1828
Sch:FA45404.1       ------------------------------   1482
Sch:KEY74888.1      ------------------------------   1410
```

Amm2

```
amm2:CDS1811        MSSKKGQRDVFRGIWELARLHTREAWVCWSPAIWGACVAAGTRDVTIDLYTLSSVLFGIW   60
Sch:KEY74889.1      MSSKTGQRDVLRGVWELARLHTREAWVCWAPAIWGACVAARTQDVYMDLYKLGSVLSGIW   60
                    ****.*****:**:***************:********** *:** :***.*.*** ***

amm2:CDS1811        ASITATHCTFCTFNDICDRNLDAHVDRCKSRPLPAGMISPPEAIVAFVAWMWASLYITRF   120
Sch:KEY74889.1      ASITATHCAFCTFNDICDRNLDAHVDRCKTRPLPAGMLSLPEAVVAFIAWMVATYYITLF   120
                    ********:********************:*******:* ***:***:*** *: *** *

amm2:CDS1811        TLGEPAVTAFVPIWVLSAVYPFMKRFIPFPQLVLGGVIGAAVFPGWAAVTRAPLDMWGGM   180
Sch:KEY74889.1      TLGQPAVMAMVPVWVLSAVYPFMKRVISFPQLVLGVVISAAVFPGWAAVTEMPLDTWGGM   180
                    ***:*** *:**:************.* ******* **.***********. *** ****

amm2:CDS1811        VARDALPLFGAGFCWVVYFDIFYATQDAPDDKAIGVLSMAVLLGKKVKLLLGVLGMLQVL   240
Sch:KEY74889.1      VAKDALPLFGAGFCWVVYFDIFYATQDAPDDKAIGVLSMAVLFGEKVKLFLGGLGVLQVL   240
                    **:***************************************:*:****:** **:****

amm2:CDS1811        FWVVAAYTARLSTFFWFAGVGVWAMNIPYHLATLKLADRHSGGKVFKANIMLGLYMSALT   300
Sch:KEY74889.1      FWAVAAYAARLSIFFWIAGVGVWAMNIPWHLVKLNLADRHSGGKVFKANILLGLYMSALT   300
                    **.****:**** ***:***********:**..*:***************:*********

amm2:CDS1811        VVELTLTRVDWYGVWQVLQEKGLEGVSLNASKGLSKEL   338
Sch:KEY74889.1      VVEMTLTRV-----------------------------   309
```

Amm3

```
amm3:CDS1810        MRKVSALAVMAASILAPQHVAATTPYDTAIANAMKGHVRLGDASFTCGVIEAVLGSNRIV   60
Sch:KEY73630.1      MRTSSLLAVAAASI----QHVYTAAYDAEIASALQGRLRIKDAGFTCSVLEAVLGSEVVV   56
                    **. * *** ****    : . *: **: **.*::*::*: **.***.*:******: :*

amm3:CDS1810        KSRDSAEYLAQEQVNYAPTCWLPAACFVSPQDVDQVSAVVRILGALKTPFSIRSGGKNLN   120
Sch:KEY73630.1      TF-EDSKYTAQEQANYSPTCWLSAACFVSPEDIDQVSAAVRIIGALQTTFSIRSGGKNLN   115
                    .  :.::* ****.**:***** *******:*:*****.***:***:* ***********

amm3:CDS1810        PGFASVDGSGVLVSLANITTLSLSADGSSLSVGAGLRWDAVYDYVVPKGLVVLGSRYTQV   180
Sch:KEY73630.1      PGFASIDESGVLLSLAKLTRLELAEDEGSLNIGPGVLWDEVYDFVVPKGLVVVGSRYNQV   175
                    *****:* ****:***::* *.*: * .**.:* *: ** ***:********:****.**

amm3:CDS1810        GVPGFLLGGGISFYTNQYGLAIDNIKSFTVVLASGKVVTASATENSDLYKALRGGNSNFG   240
Sch:KEY73630.1      GVPGFLLGGGISFYTNQYGVAIDNIKSFTVVLASGKVVVASSFKYCDLYKALKGGNSNFG   235
                    *******************:******************.**: : .******:*******

amm3:CDS1810        IVVTFELYTYPAETLSWEIRGYSADESPAILEAYAEFQLSGMQRDPLARVDINIYTDGPK   300
Sch:KEY73630.1      VVVNFELYTYPHETLHWQTRGYNPEDAGATLEAYAEFQLTGMS-DPLARVDMNIFGDRPI   294
                    :**.******* *** *: ***. ::: * *********:**. *******:**: * *

amm3:CDS1810        IIMLYNGPEPSPGVFDAFLNLTIPYNELVQPSKGNYLELQEIQSIRFDDPHVHDYGETFS   360
Sch:KEY73630.1      IIMIYNGGAPPPGTFDAFLNLTVPFEELVPPTSGTYLELQAIQAIRFINEHPHNYGETFS   354
                    ***:***  * **.********:*::*** *:.*.***** **:*** : * *:******

amm3:CDS1810        HTINETFMVEIEKLFLELMVEPSATYENFTYLWVPVAMPATVATSDNVLGVEAVEQQWHE   420
Sch:KEY73630.1      HTINGTYMAEIEQVFLELMVEPSNTYEDFSFLWVPVAMPASVARDDNVLGIEPVEQQWHE   414
                    **** *:*.***::********* ***:*::*********:** .*****:* *******

amm3:CDS1810        WVISWVNDADNDAMLELGRNITTKLTAAAEEAGTLLPYIFMNTAGGTQKVLQSFGDKNVA   480
Sch:KEY73630.1      WMIEWDNDEYNDAMLELGRNVTAVLTAATDEAGTLLPYLFMNTAGGSQPVLQSYGEENVA   474
                    *:*.* **  **********:*: ****::********:*******:* ****:*::***

amm3:CDS1810        FMKQVAKKYDPTGVFQLQNDGWLLRDV      507
Sch:KEY73630.1      FMKAVAKKYDPTAVFQRQFGGWLLRDL      501
                    *** ********.*** * .******:
```

Amm4

```
amm4:CDS13310D      MAPAKPKSYTAGSNPVNKFEGAFIVTKHAPPEGRNIHFHVRIDPHHPRLLELQKNGDKKP   60
Sch:KEY73629.1      MAPAKPKSYMAGSNPVSKFEGAFVVTKHAAPEGRNIHFHV--------------------   40
Sch:KFA74226.1      MAPAKPKSYTAGSNPVSKFEGAFVVTKHAAPEGRNIHFHVVIDPHHPRLLELLK-GDKKP   59
Sch:KFA45405.1      MAPAKPKSYTAGSNPVSKFEGAFVVTKHAAPEGRNIHFHVVIDPHHPRLLELLK-GDKKP   59
                    ********* ******.******:***** **********

amm4:CDS13310D      PTHFHPRQWEFFRVIRGSLTVEINGVPHEFVESDGEYSLPPGPHHCLYPTYGQPEGAITE   120
Sch:KEY73629.1      --------WEFFRVVRGSLTVEINGVPHQFVESDGEYALPPGPHHCLYPTDGQPKGAVVE   92
Sch:KFA74226.1      PTHFHPRQWEFFRVVRGSLTVEINGVPHQFVESDGEYALPPGPHHCLYPTDGQPKGAVVE   119
Sch:KFA45405.1      PTHFHPRQWEFFRVVRGSLTVEINGVPHQFVESDGEYALPPGPHHCLYPTDGQPKGAVVE   119
                            ******:*************:********:************ ***:**:.*

amm4:CDS13310D      FWLGATPSGSMAELDQAFFENWYGYQEDILLRGVEPDPIQVMAMFDAGDSYLSPPAWVPE   180
Sch:KEY73629.1      FWLGATPSGSMAELNQAFFENWYGYQEDILLHGVEPDPIQVMAMFDAGDSYLSPPAWVPE   152
Sch:KFA74226.1      FWLGATPSGSMAELDQAFFENWYGYQEDILLHGVEPDPIQVMAMFDAGDSYLSPPAWVPE   179
Sch:KFA45405.1      FWLGATPSGSMAELDQAFFENWYGYQEDILLHGVEPDPIQ-----------------VPK   162
                    **************:****************:********                 **:

amm4:CDS13310D      PLRHYIGKIMGVVVGRWIGGLLGYAPFFPEWTTDWPAACRQMSEHPTQKKFADLNAQEKI   240
Sch:KEY73629.1      PLRHHIGKFMGVVLGRWIGGMLGYAPFYPEWTTDWAAACRQMNLCSTQKKFAQPNAQENI   212
Sch:KFA74226.1      PLRHHIGKFMDVVLGRWIGGMLGYAPFYPEWTTDWAAACRQMNLCSTQKKFAQPNAQENI   239
Sch:KFA45405.1      PLRHHIGKFMGVVLGRWIGGMLGYAPFYPEWTTDWAAACRQMNLCSTQKKFAQPNAQENI   222
                    ****:***:*.**:******:******:******* ******.   ******: ****:*

amm4:CDS13310D      RASYLERGAQIGNEALYEEFGGYKNWLARKTK 272
Sch:KEY73629.1      RASYLERGVDIGNEALYDEHGGYENCTAKKTK 244
Sch:KFA74226.1      RASYLERGVDIGNEALYDEHGGYENCTAKKTK 271
Sch:KFA45405.1      RASYLERGVDIGNEALYDEHGGYENCMAKKTK 254
                    ********.:*******:*.***:*  *:***
```

Amm5

```
amm5:CDS13310C      ------------------------------------------------------------   0
Sch:KFA45406.1      MGRIDVHHHFIPQAYRDAFTSSADGDLSGWLLPEWTVKSTVDLMEKHRIGTAMLSITSPG   60
Sch:KEY73628        ------------------------------------------------------------   0
Sch:KFA80927.1      ------------------------------------------------------------   0


amm5:CDS13310C      ------------------------------------------------------------   0
Sch:KFA45406.1      TSVLNHDLPGANALSRAMNESAAAWRDADPNKFGLFASLPPVTATNMAAILQEVTHAFDV   120
Sch:KEY73628        ------------------------------------------------------------   0
Sch:KFA80927.1      ------------------------------------------------------------   0


amm5:CDS13310C      ------MIY--LLLVLLGAGVCLIHL------IKNSSLFSSSTSSSNPGKTKGWKSLPQP   46
Sch:KFA45406.1      PHADGVTLFTRYGRHYLGHGTFRPLWAELDSWKLRPVKDEASLLSSNQGETKDGKALPEP   180
Sch:KEY73628        ------------------MTYLLPVVALLGAGVCLSLIRSSSLLSSNQGETKDGKALPEP   42
Sch:KFA80927.1      ------------------MTYLLPVVALLGAGVCLSLIRSSSLLSSNQGETKDGKALPEP   42
                                                           .:*  *** *:**. *:**:*

amm5:CDS13310C      AGPPCLPLIGNLHQIPKTGAHRQFTEWAKTYGGIFSLKLGPSLAVVVTDRRLVREMLDRK   106
Sch:KFA45406.1      PGPPRLPVIGNIHQIPMTGAHRQFTEWSKAYGGIFSLKMGPALAVVVTDRRLVREMLDRK   240
Sch:KEY73628        PGPPRLPVIGNIHQIPMTGAHRQFTEWSKAYGGIFSLKMGPALAVVVTDRRLVREMLDRK   102
Sch:KFA80927.1      PGPPRLPVIGNIHQIPMTGAHRQFTEWSKAYGGIFSLKMGPALAVVVTDRRLVREMLDRK   102
                     *** **:***:**** **********:*:********:**:******************

amm5:CDS13310C      RAVYSARPHSYVSHDLITRGDHLLTMQYGDLWRKFRRILHPFFMESAVDKSHLALVEAEQ   166
Sch:KFA45406.1      SAIYSARPHSYVSHDLITNGAHMLTMQYGDLWRKFRRILHPYFMESAIDKVHLELVEAEQ   300
Sch:KEY73628        SAIYSARPHSYVSHDLITNGAHMLTMQYGDLWRKFRRILHPYFMESAIDKVHLELVEAEQ   162
Sch:KFA80927.1      SAIYSARPHSYVSHDLITNGAHMLTMQYGDLWRKFRRILHPYFMESAIDKVHLELVEAEQ   162
                     *:***************.* *:******************:*****:** ** ******

amm5:CDS13310C      VAMVKDFLDRPGEHMVHTKRTSNSIIMSLVFGVRTPDATTPHMRDLYDVMERWSAVMETG   226
Sch:KFA45406.1      IAMIKDFLDEPGKHTVHTKRTSNSIIMSVVFGVRTPDATTSHMRDLYNLMERWSAVMETG   360
Sch:KEY73628        IAMIKDFLDEPGKHTVHTKRTSNSIIMSVVFGVRTPDATTSHMRDLYNLMERWSAVMETG   222
Sch:KFA80927.1      IAMIKDFLDEPGKHTVHTKRTSNSIIMSVVFGVRTPDATTSHMRDLYNLMERWSAVMETG   222
                    :**:*****.**:* *************:*********** ******::***********

amm5:CDS13310C      ATPPVDIFPFLKMVPESWFGNWVQRALDVGTSMRTLYSTQKGLVLARRAGNLKGHANSHA   286
Sch:KFA45406.1      STPPVDIFPFLKMVPESWFGNWVQRGLDVGQRMKTLYSKQKGLILARRAANLKGHANG--   418
Sch:KEY73628        STPPVDIFPFLKMVPESWFGNWVQRGLDVGQRMKTLYSKQKGLILARRAANLKGHANG--   280
Sch:KFA80927.1      STPPVDIFPFLKMVPESWFGNWVQRGLDVGQRMKTLYSKQKGLILARRAANLKGHANG--   280
                    :************************.****  *:****.****:*****.*******.
```

```
amm5:CDS13310C      NNGGAAAAERPTTLMDEVLDQQDKLQLTKHQQDFVGGVLMEGGSDTVSTMMLVVLQALCL  346
Sch:KFA45406.1      ---------QPTTLMDEVLDQQDKLQLTNHQQDFLGGVLMEGGSDTVSTMMLVVLQALCL  469
Sch:KEY73628        ---------QPTTLMDEVLDQQDKLQLTNHQQDFLGGVLMEGGSDTVSTMMLVVLQALCL  331
Sch:KFA80927.1      ---------QPTTLMDEVLDQQDKLQLTNHQQDFLGGVLMEGGSDTVSAMMLVVLQALCL  331
                             :******************:*****:*************:***********

amm5:CDS13310C      NPDIVQRAQRDIDAVLDEDGTPRWEHRDKLPYITQIVKEAFRWRPVTPLGFPHALLSAEQ  406
Sch:KFA45406.1      NPDIVERARRDIDAVVGEDSTPRWEHHDKLPYITQIVKEAMRWRPVTPLEFPHALLSAEQ  529
Sch:KEY73628        NPDIVERARRDIDAVVGEDSTPRWEHHDKLPYITQIVKEAMRWRPVTPLGFPHALLSAEQ  391
Sch:KFA80927.1      NPDIVERARRDIDAVVGEDSTPRWEHHDKLPYITQIVKEAMRWRPVTPLGFPHALLSAEQ  391
                    *****:**:******:.**.******:*************:******** **********

amm5:CDS13310C      GGQGDVVDGRFWLPPGTPVFLNVWGLHQDCKDPDRFDPDRYDGQTKTSAEYAASSEYELR  466
Sch:KFA45406.1      GGKGDVVDGRFYLSPGTTVFLNVWGIHQDCEDPNRFDPDRYDGRTKTSAEYAASSEYELR  589
Sch:KEY73628        GGKGDVVDGRFYLSPGTTVFLNVWGIHQDCEDPDRFDPDRYDGRTKTSAEYAASSEYELR  451
Sch:KFA80927.1      GGKGDVVDGRFYLSPGTTVFLNVWGIHQDCKDPDRFDPDRYDGRTKTSAEYAASSEYELR  451
                    **:********:* *** *******:****:**:*********:****************

amm5:CDS13310C      DHYVFGAGRRICPGIHLAEREMFLGTAKLLWGFNIEQARDEMGNVIPIDTNPVTGYTEGF  526
Sch:KFA45406.1      DHYVFGAGRRICPGIHLAEREMFLGTAKLLWGFNIEQARDAKGVLIPIDTDPVTGYSEGF  649
Sch:KEY73628        DHYVFGAGRRICPGIHLAEREMFLGTAKLLWGFNIEQARDDKGGLIPIDTDPVTGYSEGF  511
Sch:KFA80927.1      DHYVFGAGRRICIEQPKNNTQL--------------------------------------  473
                    ************      : ::

amm5:CDS13310C      LVCPKDFACNITPRSERRAATIVREFEEARENVFSLYE----      564
Sch:KFA45406.1      LVCPKDFDCSITPRSERLTATILREFVEASKECLFTLQVMQG      691
Sch:KEY73628        LVCPKDFDCSITPRSERRTATILREFVEASKNVFSRYK----      549
Sch:KFA80927.1      ------------------------------------------      473
```

Amm6

```
Amm6:CDS13310B      ------------------------------------------------------------  0
Sch:KEY73627.1      ------------------------------------------------------------  0
Sch:KFA80926.1      MEAERDPVKVDAETAPPTSTSSSLPDQRESKKAAVEAEKDVDPNEYPTGIRLVAVILALV  60

Amm6:CDS13310B      ------------------------------------------------------------  0
Sch:KEY73627.1      ------------------------------------------------------------  0
Sch:KFA80926.1      LCVFLMPLDTTILATAIPRITADFGLADVSWYASVFFFVVAGFQSSWGKAFRYFPLKPAL  120

Amm6:CDS13310B      ------------------------------------------------------------  0
Sch:KEY73627.1      ------------------------------------------------------------  0
Sch:KFA80926.1      IVSLIIFEVGSIVAATAQSSVALIIGRAVTGLGAAGMSSGAFLVAGLIGPPRKRPIYIGI  180

Amm6:CDS13310B      ------------------------------------------------------------  0
Sch:KEY73627.1      ------------------------------------------------------------  0
Sch:KFA80926.1      IGVSASIGAVSGPLIGGALTTDLSWRWCFWINLPLGGAAVLFLLFFFRTPPSWKPKQETF  240

Amm6:CDS13310B      ------------------------------------------------------------  0
Sch:KEY73627.1      ------------------------------------------------------------  0
Sch:KFA80926.1      AKKLWHLDPVGVALVMGLSIAYTLALQYAGNGESWGSGRVAGLIVGFVVILVGFGVWEWY  300

Amm6:CDS13310B      ------------------------------------------------------------  0
Sch:KEY73627.1      ------------------------------------------------------------  0
Sch:KFA80926.1      QGDNAMVPSRIARQRDLTVGCAVTFFLSGGFFISEYYLPIYFQAIDGVSARQSGINYLPT  360

Amm6:CDS13310B      ------------------------------------------------------------  0
Sch:KEY73627.1      ------------------------------------------------------------  0
Sch:KFA80926.1      IIASGVAILVFGGIMSVTGVVTPYLHASGVISIIAAGLLYMLDLDTSTARWIGYQILWGF  420

Amm6:CDS13310B      ------------------------------------------------------------  0
Sch:KEY73627.1      ------------------------------------------------------------  0
Sch:KFA80926.1      GSGLGMNIPILIGQDRVDPADMSVATSLILLFQTLGGSFLVSAANAGFSSTLRQSLVMEA  480

Amm6:CDS13310B      ------------------------------------------------------------  0
Sch:KEY73627.1      ----------------------------------------------------------MG  2
Sch:KFA80926.1      PTLSPEEVIAAGAYDLQSNFGGDVLHGVQVSYMDALQVVFAVMIASRGVSFLAGLGMSWK  540
```

```
Amm6:CDS13310B      ----------MHNENDQTFVNSADGDLSGWILPEWTVKSTLDLMGKHRIGTSILSITSPG  50
Sch:KEY73627.1      RIDV--HHHFIPQAYRDAFTSSADGDLSGWLLPEWTVKSTVDLMEKHRIGTAMLSITSPG  60
Sch:KFA80926.1      KLDAEKMKNPGNPAYRDAFTSSADGDLSGWLLPEWTVKSIVDLMEKHRIGTAMLSITSPG  600
                                    ::*..*********:******** :*** ******::*******

Amm6:CDS13310B      TSILNDDLPGANALCRAINKSAAALRDTHSSKLGFFATLPPVTTTNMAAVLDEVTYALDV  110
Sch:KEY73627.1      TSVLNHDLPGANALSRAMNESAAAWRDADPNKFGLFASLPPVTATNMAAVLQEVTHAFDV  120
Sch:KFA80926.1      TSVLNHDLPGANALSRAMNESAAAWRDADPNKFGLFASLPPVAATNMAAVLQEATHAFDV  660
                    **:**.********.**:*:**** **:. .*:*:**:****::*******:*.*:*:**

Amm6:CDS13310B      LHADGVTLFTRYGPHYLGHETLRPLWAELDKHKAVVFIHPTHSVGHDIVSSALPQPVIDY  170
Sch:KEY73627.1      LHADGVTLFTRYGPHYLGHETFRPLWAELDSRKAVVFIHPTHSVGHDTTSSAAPQPLIDY  180
Sch:KFA80926.1      LHVDGVTLFTRYGPHYLGHETFRPLWAELDSRKAVVFIHPTHSVGHDTTSSAAPQPLIDY  720
                    **.******************:********.:*************** .*** ***:***

Amm6:CDS13310B      PHETTRTAVDLIQQGVMANFPAVKVILSHAGGTLPYLAMRAAHLAADARFSTLSAGEFLD  230
Sch:KEY73627.1      PHETTRTAVDMIQQGVVVDFPAVKVILSHAGGTLPYLAMRAAHLAVDARFSTLPAEDFLA  240
Sch:KFA80926.1      PHETTRTAVDMIQQGVVVDFPAVKVILSHAGGTLPYLAMRAAHLAVDARFSTLPAEDFLA  780
                    **********:*****:.:**************************.******* * :**

Amm6:CDS13310B      RARSFYFDIALSSNPIQLDLLLGFAKPGHVLYGSDFPYAPGKTIGTFVEALDAYEEKLDE  290
Sch:KEY73627.1      RARTFYFDLALSSNPSQLDLVLAFAKPGHVLYGNDFPYAPSKTIGTFAEALDAYEEKLDE  300
Sch:KFA80926.1      RARTFYFDLALSSNPSQLDLVLAFAKPGHVLYGSDFPYAPSKTIGTFAEALDAYEEKLDE  840
                    ***:****:****** ****:*.**********.******.******.************

Amm6:CDS13310B      ETKYSITRGAALQLFPRLRGAEGDA       315
Sch:KEY73627.1      ETKYSIMRGGALQLFPRLRVEGDV-       324
Sch:KFA80926.1      ETKYFITRGGALQLFPRLRVEGDV-       864
                    **** * **.*********    .
```

Amm7

```
amm7:CDS13310A      MAGLVNSSA--ATAAPSWLGVLRDSAASTGLVAPQFIICALLLTTIILLFPPRSLFRRAL  58
Sch:KFA53562.1      MAVPTNSSATATAPPSSLLRVLRDSTGSTGLVAPQFIICALLLTTVIFLFPNVI------  54
Sch:KEY73623.1      MAVPTNSSATATAPPSSLLRVLRDSTGSTGLVAPQFIICALLLTTVIFLFPVGSLFRRAL  60
                    **  .****  ::   * * *****:.******************:*:***

amm7:CDS13310A      YLVQLYLTACAFVAPLPPGAPQADLYAAGLLIGGWAARILDRVYMVQEPEKAFLRKGVDD  118
Sch:KFA53562.1      ------------------------------------------------------------  54
Sch:KEY73623.1      YPVQLG------------------------------------------------------  66


amm7:CDS13310A      GPNGPETYGPLRKFNWAFEMIFSQRGVGWNWQVGGVPRPDYTTRWGFVANRVFRSLYTMF  178
Sch:KFA53562.1      ------------------------------------------------------------  54
Sch:KEY73623.1      ------------------------------------------------------------  66


amm7:CDS13310A      LVHMVSVLADVILAIDQSGGEGTISSGLAAVLRHPLFLRAYVTAGWLIVVYGHVALPENA  238
Sch:KFA53562.1      ------------------------------------------------------------  54
Sch:KEY73623.1      ------------------------------------------------------------  66


amm7:CDS13310A      ISIVTVATGVFGRWSDPKLWPPMFNKMSEAYTLRRYWGKYWHAMLRRTTNAPGEFLLQEI  298
Sch:KFA53562.1      -SIVTVSTGMFGRWSDPKLWPPMFNSMSEAYSLQRYWGKYWHMMLRRTTDAPGFFLQQEI  113
Sch:KEY73623.1      LIAFTVSTGMFGRWSDPKLWPPMFNSMSEAYSLRRYWGKYWHMMLRRTTDAPGFFLQQEI  126
                       .**:**:***************.*****:*:******** ******:*** ** ***

amm7:CDS13310A      PALRKPKSQFVRLARRYGLLFLSFAVSGFIHAAGSYMVTRDYPEGWSDGGAMKYFLVQPV  358
Sch:KFA53562.1      PVLRNPKNQVVRLVRRYGLLFLSFAVSGLIHAAGSYMVTRDFPEGWSDGGAMVYFLVQPA  173
Sch:KEY73623.1      PVLRNPKNQVVRLVRRYGLLFLSFAVSGLIHAAGSYMVTRDFPEGWSDGGAMVYFLVQPA  186
                    *.**:**.*.***.**************:************:********** ******.

amm7:CDS13310A      AIVLEDALYIALGVPDDGNPGLIRRLVGYAYVTAWWLWCFPTLKVAPLAAAHRLEGWEQG  418
Sch:KFA53562.1      FILVEDTLFMALGVPDDGNPALLRRLFGYAYVTAWWLWCFPTLKVAPLAAAHRLDGWDQD  233
Sch:KEY73623.1      FILVEDTLFMALGVPDDGNPALPRRLFGYAYVTAWWLWCFPTLKVAPLAAAHRLDGWDQD  246
                     *::**:*::**********.* ***.***************************:**:*.

amm7:CDS13310A      GLLASVVACKELADAYPVNFARDLWHTLGRA  449
Sch:KFA53562.1      SWRASVVACKELADAYPFNPARNLWQALGRS  264
Sch:KEY73623.1      SWRASVVACKELADAYPFNPARNLWQALGRS  277
                    .  **************.* **:**::***:
```

Amm8

```
amm8:CDS13309       MFEILMQGYTVIGVELLVLTLVIYHIVRAIYLIYFSPLSVFPGSPWAALGEYWEAWHNIG   60
Sch:KFA80928.1      -----------------------------------------------MGGSRRIAWHNIG   13
Sch:KEY73624.1      MFQTLMQSNPGTGVVVVVLSFVVYHVVRAIYLIYFSPLSVFPGSPWAALGEYWEAWHNIG   60
Sch:KFA45408.1      ------------------------------------------------------------   0


amm8:CDS13309       SSPGKRGQTLFLLERMHRDPKYGSAIRMGPNEVHIYDPRFFHQLYSLNTRFYKDASMHKV   120
Sch:KFA80928.1      SSPGKRGQTLFLLETMHHDPKYGSAIRMGPNEVHVYDPRFFHQLYSLNTRFYKDESMHKV   73
Sch:KEY73624.1      SSPGKRGQTLFLLETMHHDPKYGSAIRMGPNEVHVYDPRFFHQLYSLNTRFYKDESMHKV   120
Sch:KFA45408.1      ---------------------------MGPNEVHVYDPRFFHQLYSLNTRFYKDESMHKG   33
                                               *******:******************* ****

amm8:CDS13309       LGAPASTLAETDPIKHKARRQPLESLFSRQSILKLQPMVLSKIDFACQRFDELYQAGKPV   180
Sch:KFA80928.1      LGAPSSTLAETDPVKHRARRQPLESLFSRQSILRLEPTVLSKIDFACQRFDELYKAGKPV   133
Sch:KEY73624.1      LGAPSSTLAETDPVKHRARRQPLESLFSRQSILRLEPTVLSKIDFACQRFDELYKAGKPV   180
Sch:KFA45408.1      LGAPSSTLAETDPVKHRARRQPLESLFSRQSILRLEPTVLSKIDFACQRFDELYKAGKPV   93
                    ****:********:**:****************:*:* ****************:*****

amm8:CDS13309       RAEWALKSLSFDIVSEFCFGSSLGALYDDDFTSDPVRVFRAYLHSLHIIKAFPLVRTISQ   240
Sch:KFA80928.1      RAEWAFKSLSFDIVSEFCFGSGPGALHDDDFTSDPVRVFRAYLHSLHIIKAFPLVRTISQ   193
Sch:KEY73624.1      RAELAFKSLSFDIVSEFCFGSGLGALHDDDFTSDPVRVFRAYLHCLHIIKAFPLVRTISQ   240
Sch:KFA45408.1      RAEWAFKSLSFDIVSEFCFGSGLGALHDDDFTSDPVRVFRAYLHSLHIIKAFPLVRTISQ   153
                    *** *:***************. ***:*****************.***************

amm8:CDS13309       SLPLWLARLASKTVARAKELEILVRGRVDTFVDAYEHGEKPSFPTAMERLLQAGEDLKAP   300
Sch:KFA80928.1      SLPLWLARAVSKTVARAKELEILVRGRVDTFVDAYENGEKPSFPTAMERLLQAGEDLKAP   253
Sch:KEY73624.1      SLPLWLARAVSKTVARAKELEILVRGRVDTFVDAYENGEKPSFPTAMERLLQAGEDLKAP   300
Sch:KFA45408.1      SLPLWLARAVSKTVARAKELEILVRGRVDTFVDAYENGEKPSFPTAMERLLQAGEDLKAP   213
                    ******** .**************************:***********************

amm8:CDS13309       GAESIPWSRDYLRDEVLTMISAGTDTTGISALVALYNVVANKAIQARLLAELMTVMPGPN   360
Sch:KFA80928.1      GAADIPWSRDYLRDEVLTMISAGTDTTGISTLVALYYVVANKDIQARLLAELKTVMPGPI   313
Sch:KEY73624.1      GAADIPWSRDYLRDEVLTMISAGTDTTGISTLVALYYVVANKDIQARLLAELKTVMPGPI   360
Sch:KFA45408.1      GAADIPWSRDYLRDEVLTMISAGTDTTGISTLVALYYVVANKDIQARLLAELKTVMPGPI   273
                    ** .**************************:***** ***** ********* ******

amm8:CDS13309       DTASFQVLEKLPYLTAVIKEGLRFASPAASRTPRLVPKGGTTLPDGRFIPGGTRVGMAIY   420
Sch:KFA80928.1      DAASFQVLEKLPYLTAVIKEGLRVASPAASRTPRLVPKGGTTLPDGRFLPGGTRVGMAIY   373
Sch:KEY73624.1      DAASFHVLEKLPYLTAVIKEGLRVASPAASRTPRLVPKGGTTLPDGRFLPGGTRVGMAIY   420
Sch:KFA45408.1      DAASFQVLEKLPYLTAVIKEGLRVASPAASRTPRLVPKGGTTLPDGRFLPGGTRVGMAIY   333
                    *:***:*****************.************************:***********

amm8:CDS13309       HVHYNSDIFPEPRKFMPERWLDAENGGAMPERLPEMNRFMVAFSKGTRACIGINLANMEL   480
Sch:KFA80928.1      HVHYNEDIFPEPKRFMPERWLDADNGGMMPERVPEMQRFMVAFSKGTRACIGINLAYMEL   433
Sch:KEY73624.1      HVHYNEDIFPEPKRFMPERWLDADNGGMMPERVPEMQRFMMAFSKGTRACIGINLAYMEL   480
Sch:KFA45408.1      HVHYNEDIFPEPKRFMPERWLDADNGGMMPERVPEMQRFMVAFSKGTRACIGINLAYMEL   393
                    *****.******::*********:*** ****:***:***:*************** ***

amm8:CDS13309       YLTLAHLIRRFDLQT---DTTDEDMKWDDMVVAWFHGEFTFMPRRRME       525
Sch:KFA80928.1      YLLFVWTSKDAHDTPAGEERDDGNLKLT--------------------       461
Sch:KEY73624.1      YLAVAHFIRRFDMQT---DTTDEDMKWDDMVVAWFHGEFKFMARRRTE       525
Sch:KFA45408.1      YLAVAHFIRRFDMQT---DTTDEDMKWDDMVVAWFHGEFKFMARRRTE       438
                    ** ..   :  .      :  * ::*
```

Amm9

```
amm9:CDS13308       MGVVQPAIPATPSGTTLAGKTVIVTGGNSGIGLEACRQMLLLGVSRLIIASRTVSNGQLA   60
Sch:KEY73625.1      MGIVQPAIPPTPSGTTLVGQTVIVTGGNAGIGLEACRQLLLLGVSRLIVASRSVSNGQLA   60
Sch:KFA45409.1      MGIVQPAIPPTPSGTTL---------------------LLLLGVSRLIVASRSVSNGQLA   39
                    **:****** *******                     :*********:***:*******

amm9:CDS13308       ISALRADKDVKRENPGAELQVFQLDLSDYVSGLKFVERVKKEVAELDILVNNGGQVELKY   120
Sch:KEY73625.1      VSALRADKDVARENPVAQLLVFQLDLSDYASGLRFVERVKKEVPELDILVNNGGQVELQY   120
Sch:KFA45409.1      VSALRADKDVARENPVAQLLVFQLDLSDYASGLRFVERVKKEVPELDILVNNGGQVELQY   99
                    :********* **** *:* *********.***:********* **************:*

amm9:CDS13308       ETAPTGHEKNMQVNCYTHMLIALELVPLLRATAKVRGVPSRITFTGSNTQLYQETLTKDP   180
Sch:KEY73625.1      ETAPTGHEKNMQVNCYTHMLITLELLPLLSATAKLRGAPSRVTFTGSITQVFQETLTKEP   180
Sch:KFA45409.1      ETAPTGHEKNMQVNCYTHMLITLELLPLLSATAKLRGAPSRVTFTGSITQVFQETLTKEP   159
                    *********************:***:*** ****:**.***:***** **::******:*
```

```
amm9:CDS13308       VGPGETVLGHWDDPKHFSNLFRYADSKLCVSAYCRKLAQLVSPDEVIEPEPRDANVHAVR  240
Sch:KEY73625.1      IGPGESVLGHWDDPKHFSNLFRYADTKLCVSAYCRKLAQLVSADEVIINDFCPGLV---R  237
Sch:KFA45409.1      IGPGESVLGHWDDPKHFSNLFRYADTKLCVSAYCRKLAQLVSADEVIVSDFCPGLV---R  216
                    :****:*******************:**************** ****  :   . *   *

amm9:CDS13308       AQGDRK--------------EFAGRREDAGIRGGGSGEGDAWNIL--E---PQSGASVSV  281
Sch:KEY73625.1      NKGLDRNLNPAMQILMQCVRQLIGRSIPDAAR------ALVYAAVVVGKETHRTFLNHNQ  291
Sch:KFA45409.1      NKGLDRNLNPAMQILMQCVRQLIGRSIPDAAR------ALVYAAVVVGKETHGTFLNHNQ  270
                     :*  :              :: **    . *      . .:  :        :  . .

amm9:CDS13308       QFVGAAILDKPEGKKFLEDLWKETVEDLAKIDPGLGMYVAA       322
Sch:KEY73625.1      VHPGAAILDTPERKKFTDDLWKETAEDLAKIDRALGMYVAA       332
Sch:KFA45409.1      VHPGAAILDTPEGKKFTDDLWKETAEDLAKIDRALGMYVAA       311
                     . ******.** *** :******.******* .*******
```

Amm10

```
amm10:CDS13307      MEAESDLMKAAGVTASAPTSSLPPSSGPDEKVLETGVLEAAQETDSEQYPTGVRLVAVIV  60
Sch:KEY73626.1      MEAERDPVKVDAETAP---PTSTSSSLPDQRESKKAAVEAEKDVDPNEYPTGIRLVAVIL  57
Sch:KFA80926.1      MEAERDPVKVDAETAP---PTSTSSSLPDQRESKKAAVEAEKDVDPNEYPTGIRLVAVIL  57
Sch:KFA45407.1      MEAERDPVKVDAETAP---PTSTSSSLPDQRESKKAAVEAEKDVDPNEYPTGIRLVAVIL  57
                    **** * :*. . **     :   ** **::  :...:** ::.* ::****:******:

amm10:CDS13307      ALAICVFLMPLDTTILATAIPKITNDFGLSDLSWYASVYLMTVAGFQSSWGKAFRYFPLK  120
Sch:KEY73626.1      ALVLCVFLMPLDTTILATAIPRITADFGLADVLWYASVFFFVVAGFQSSWGKAFRYFPLK  117
Sch:KFA80926.1      ALVLCVFLMPLDTTILATAIPRITADFGLADVSWYASVFFFVVAGFQSSWGKAFRYFPLK  117
Sch:KFA45407.1      ALVLCVFLMPLDTTILATAIPRITADFGLADVSWYASVFFFVVAGFQSSWGKAFRYFPLK  117
                    **.:*****************:** ****:*: *****:::.******************

amm10:CDS13307      PALLAAVVIFELGSVVAATARSSIVLIIGRAISGLGAAGMSSGCFLVAGLIGPPQKRPIY  180
Sch:KEY73626.1      PALIVSLIIFEVGSIVAATAQSSVALIIGR-------------------------ARPIY  152
Sch:KFA80926.1      PALIVSLIIFEVGSIVAATAQSSVALIIGRAVTGLGAAGMSSGAFLVAGLIGPPRKRPIY  177
Sch:KFA45407.1      PALIVSLIIFEVGSIVAATAQSSVALIIGRAVTGLGAAGMSSGAFLVAGLIGPPRKRPIY  177
                    ***:.:::***:**:*****:**:.*****                          ****

amm10:CDS13307      IGIIGVAASVGAVTGPLIGGALTTGLSWRWCFYINLPVGGVAGLFLLIFFRTPASWKAKD  240
Sch:KEY73626.1      IGIIGVSASIGAVSGPLIGGALTTDLSWRWCFWINLPLGGAAVLFLLFFFCTPPSWKPKQ  212
Sch:KFA80926.1      IGIIGVSASIGAVSGPLIGGALTTDLSWRWCFWINLPLGGAAVLFLLFFFRTPPSWKPKQ  237
Sch:KFA45407.1      IGIIGISASIGAVSGPLIGGALTTDLSWRWCFWINLPLGGAAVLFLLFFFRTPPSWKPKQ  237
                    *****::**:***:**********.*******:****:**.* ****:** ** *** *:

amm10:CDS13307      ETLMAKLWHLDPIGVILVMAFSISFTLALQYAGNGESWGSGKVAGLLVGFVAITAVFALW  300
Sch:KEY73626.1      ETFAKKLWHLDPVGVALVMGLSIAYTLALQYAGNGESWGSGRVAGLIVGFVVILVGFGVW  272
Sch:KFA80926.1      ETFAKKLWHLDPVGVALVMGLSIAYTLALQYAGNGESWGSGRVAGLIVGFVVILVGFGVW  297
Sch:KFA45407.1      ETFAKKLWHLDPVGVALVMGLSIAYTLALQYAGNGESWGSGRVAGLIVGFVAILVGFGVW  297
                    **:  *******:** ***.:**::****************:****:****.* . *.:*

amm10:CDS13307      EWYQGEHAMIPGRVARQRDVAVSCVVTFFLSGGFFIVEYYLPTYFQAVEGVNALTSGIHY  360
Sch:KEY73626.1      EWYQGDNAMVPSRIARQRDLAVGCAVTFFLSGGFFISEYYLPIYFQAIDGVSARQSGINY  332
Sch:KFA80926.1      EWYQGDNAMVPSRIARQRDLTVGCAVTFFLSGGFFISEYYLPIYFQAIDGVSARQSGINY  357
Sch:KFA45407.1      EWYQGDNAMVPSRIARQRDLAVGCAVTFFLSGGFFISEYYLPIYFQAIDGVSARQSGINY  357
                    *****::**:*.*:*****::*.*.*********** ***** ****::**.*  ***:*

amm10:CDS13307      LPTIIASGLAIMVFGGIMSSTGVVTPYLHVSGVISTIAAGLLYMLDLDTSMARWIGYQIL  420
Sch:KEY73626.1      LPTIIASGVAILVFGGIMSVTGVVTPYLHASGVISIIAAGLLYMLDLDTSTARWIGYQIL  392
Sch:KFA80926.1      LPTIIASGVAILVFGGIMSVTGVVTPYLHASGVISIIAAGLLYMLDLDTSTARWIGYQIL  417
Sch:KFA45407.1      LPTIIASGVAILVFGGIMSVTGVVTPYLHASGVISIIAAGLLYMLDLDTSTARWIGYQIL  417
                    ********:**:******* *********.***** ************** *********

amm10:CDS13307      WGVGSGMGMNVPILIGQTRVETSDMSVVTALVLLFQTLGGSFMVSAGQAGFASTLRRSIL  480
Sch:KEY73626.1      WGFGSGLGMNIPILIGQDRVDPADMSVATSLILLFQTLGGSFLVSAANAGFSSTLRQSLV  452
Sch:KFA80926.1      WGFGSGLGMNIPILIGQDRVDPADMSVATSLILLFQTLGGSFLVSAANAGFSSTLRQSLV  477
Sch:KFA45407.1      WGFGSGLGMNIPILVGQDRVDPADMSVATSLILLFQTLGGSFLVSAANAGFSSTLRQSLV  477
                    **.***:***:***:** **: :****.*:*:**********:***.:***:****:*::

amm10:CDS13307      TEAPTVSPDLVIATGAADLQNTFGPDVLHGVLEAYVDALKVVFAVATASRGVSFLVGLFM  540
Sch:KEY73626.1      MEAPTLSPEEVIAAGAYDLHSNFGGDVLHGVQVSYMDALQVVFAVMIASRGVSFLAGLGM  512
Sch:KFA80926.1      MEAPTLSPEEVIAAGAYDLQSNFGGDVLHGVQVSYMDALQVVFAVMIASRGVSFLAGLGM  537
Sch:KFA45407.1      MEAPTLSPEEVIAAGAYDLQSNFGGDVLHGVQVSYMDALQVVFAVMIASRGVSFLAGLGM  537
                     ****:**: ***:** **:..** ******  :*:***:*****  ********.** *

amm10:CDS13307      SWKKLDTDKLKNLGDPVVDGKREEE-GGEVKGKEADL-----------------------  576
Sch:KEY73626.1      SWKKLDAEKMKNPGNPV-------------------------------------------  529
Sch:KFA80926.1      SWKKLDAEKMKNPGNPAYRDAFTSSADGDLSGWLLPEWTVKSIVDLMEKHRIGTAMLSIT  597
Sch:KFA45407.1      SWKKLDAEKMKNPGNPV-------------------------------------------  554
                    ******::*:** *:*.
```

```
amm10:CDS13307      ------------------------------------------------------------   576
Sch:KEY73626.1      ------------------------------------------------------------   529
Sch:KFA80926.1      SPGTSVLNHDLPGANALSRAMNESAAAWRDADPNKFGLFASLPPVAATNMAAVLQEATHA   657
Sch:KFA45407.1      ------------------------------------------------------------   554

amm10:CDS13307      ------------------------------------------------------------   576
Sch:KEY73626.1      ------------------------------------------------------------   529
Sch:KFA80926.1      FDVLHVDGVTLFTRYGPHYLGHETFRPLWAELDSRKAVVFIHPTHSVGHDTTSSAAPQPL   717
Sch:KFA45407.1      ------------------------------------------------------------   554

amm10:CDS13307      ------------------------------------------------------------   576
Sch:KEY73626.1      ------------------------------------------------------------   529
Sch:KFA80926.1      IDYPHETTRTAVDMIQQGVVVDFPAVKVILSHAGGTLPYLAMRAAHLAVDARFSTLPAED   777
Sch:KFA45407.1      ------------------------------------------------------------   554

amm10:CDS13307      ------------------------------------------------------------   576
Sch:KEY73626.1      ------------------------------------------------------------   529
Sch:KFA80926.1      FLARARTFYFDLALSSNPSQLDLVLAFAKPGHVLYGSDFPYAPSKTIGTFAEALDAYEEK   837
Sch:KFA45407.1      ------------------------------------------------------------   554

amm10:CDS13307      ---------------------------      576
Sch:KEY73626.1      ---------------------------      529
Sch:KFA80926.1      LDEETKYFITRGGALQLFPRLRVEGDV      864
Sch:KFA45407.1      ---------------------------      554
```

Fig. S2. Identification of Acremeremophilane N in *Trichoderma virens* G2 and its absence in the knockout mutant Δ*amm6.*

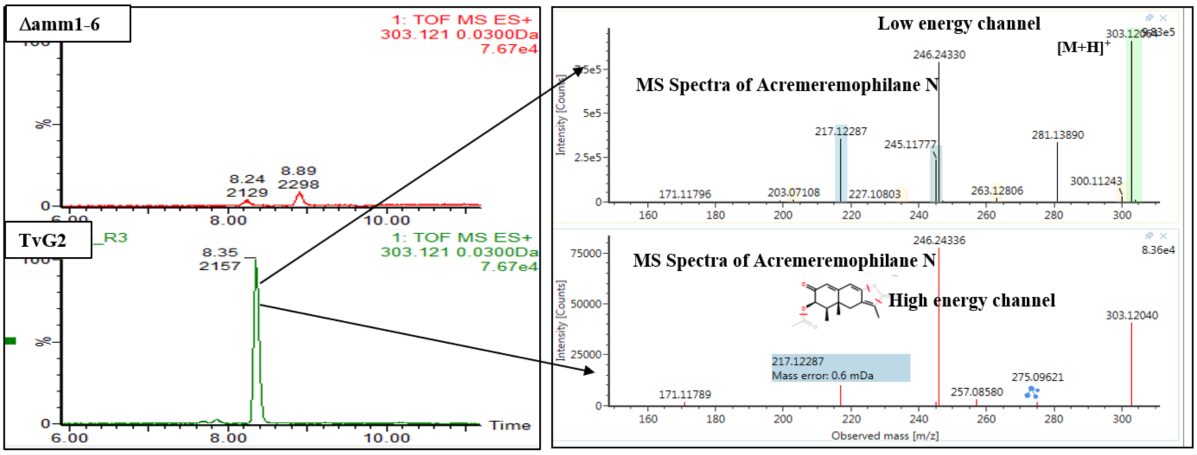

Fig. S3. Identification of Acremeremophilane K in *Trichoderma virens* G2 and its absence in the knockout mutant Δ*amm6.*

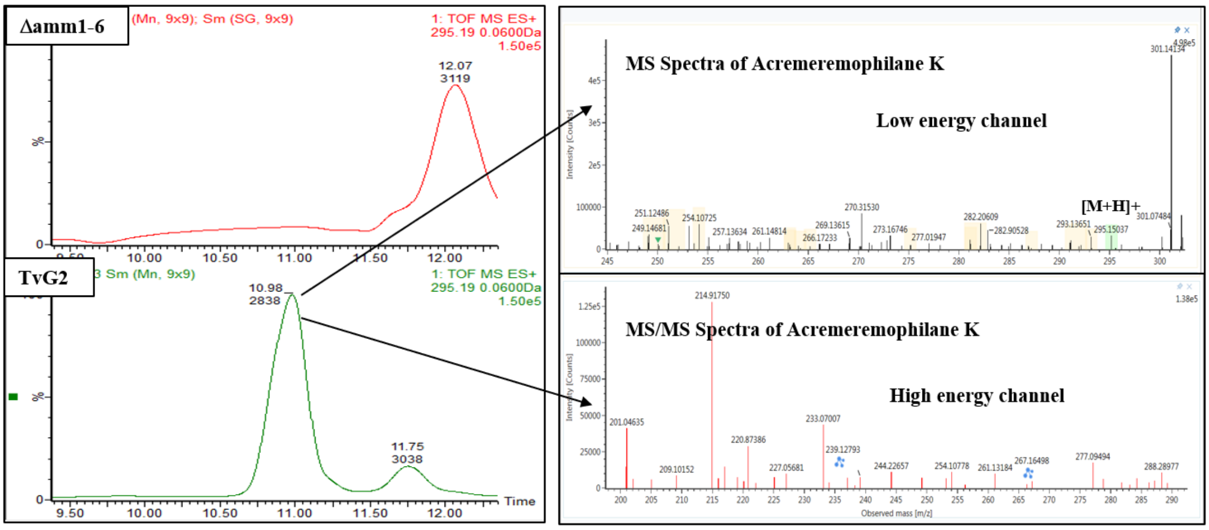

Fig. S4. Identification of Acremeremophilane I in *Trichoderma virens* G2 and its absence in the knockout mutant Δ*amm6.*

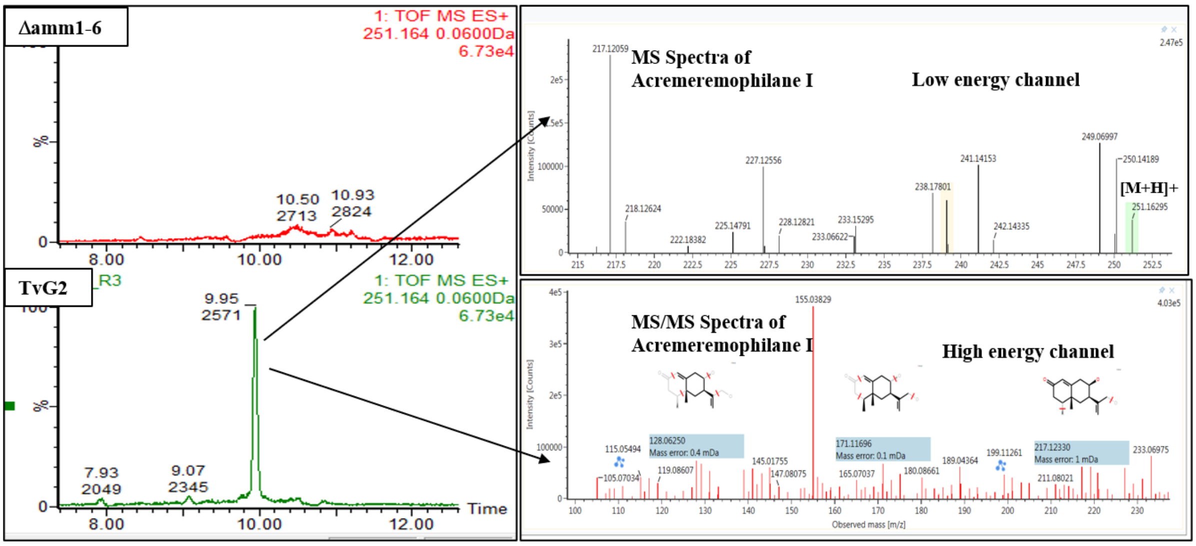

Fig. S5. Identification of Acremeremophilane O in *Trichoderma virens* G2 and its absence in the knockout mutant Δ*amm6.*

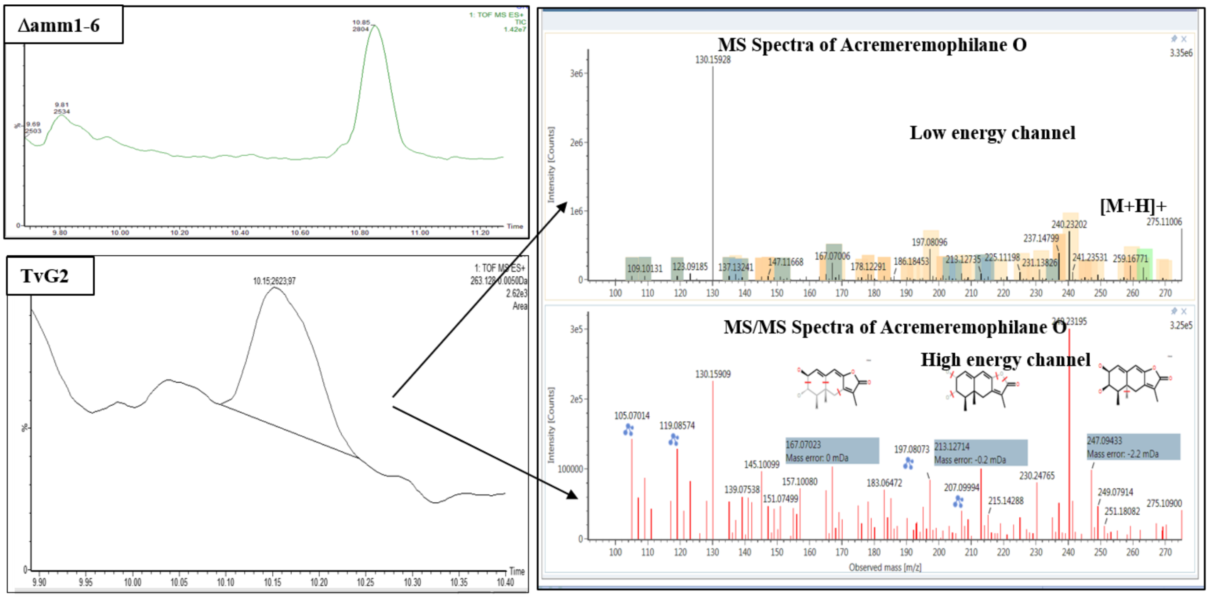

Supplement: Supplemental file 1 — Supplemental material. Download spectrum.01793-22-s0001.pdf, PDF file, 0.8 MB [file spectrum.01793-22-s0001.pdf]
